# Metabolomics Reveals That Bisphenol Pollutants Impair Protein Synthesis-Related Pathways in *Daphnia magna*

**DOI:** 10.3390/metabo11100666

**Published:** 2021-09-29

**Authors:** Erico A. Oliveira Pereira, Lisa M. Labine, Sonya Kleywegt, Karl J. Jobst, André J. Simpson, Myrna J. Simpson

**Affiliations:** 1 Environmental NMR Centre and Department of Physical and Environmental Sciences, University of Toronto Scarborough, 1265 Military Trail, Toronto, ON M1C 1A4, Canada; erico.pereira@mail.utoronto.ca (E.A.O.P.); lisa.labine@mail.utoronto.ca (L.M.L.); andre.simpson@utoronto.ca (A.J.S.); 2 Department of Chemistry, University of Toronto, 80 St. George St., Toronto, ON M5S 3H6, Canada; 3 Technical Assessment and Standards Development Branch, Ontario Ministry of the Environment, Conservation and Parks, Toronto, ON M4V 1M2, Canada; sonya.kleywegt@ontario.ca; 4 Department of Chemistry, Memorial University of Newfoundland, St. John’s, NL A1B 3X7, Canada; karl.jobst@mun.ca

**Keywords:** ecotoxicology, metabolomics, bisphenol pollutants, bisphenol A (BPA), bisphenol F (BPF), bisphenol S (BPS)

## Abstract

Bisphenols are used in the production of polycarbonate plastics and epoxy resins. Bisphenol A (BPA) has been widely studied and is believed to act as an endocrine disruptor. Bisphenol F (BPF) and bisphenol S (BPS) have increasingly been employed as replacements for BPA, although previous studies suggested that they yield similar physiological responses to several organisms. *Daphnia magna* is a common model organism for ecotoxicology and was exposed to sub-lethal concentrations of BPA, BPF, and BPS to investigate disruption to metabolic profiles. Targeted metabolite analysis by liquid chromatography-tandem mass spectrometry (LC-MS/MS) was used to measure polar metabolites extracted from *D. magna*, which are linked to a range of biochemical pathways. Multivariate analyses and individual metabolite changes showed similar non-monotonic concentration responses for all three bisphenols (BPA, BPF, and BPS). Pathway analyses indicated the perturbation of similar and distinct pathways, mostly associated with protein synthesis, amino acid metabolism, and energy metabolism. Overall, we observed responses that can be linked to a chemical class (bisphenols) as well as distinct responses that can be related to each individual bisphenol type (A, F, and S). These findings further demonstrate the need for using metabolomic analyses in exposure assessment, especially for chemicals within the same class which may disrupt the biochemistry uniquely at the molecular-level.

## 1. Introduction

Bisphenol A (4,4′-(Propane-2,2-diyl)diphenol; BPA) is an organic synthetic compound used in the manufacturing of polycarbonate plastics and epoxy resins [1,2]. With an estimated global annual production of 8 million tons per year [3], BPA can be found in a multitude of products ranging from can coatings, dental fillings, thermal papers to electronics, medical devices and household appliances [1,3]. BPA exposure is believed to induce endocrine disruption in several organisms, either due to binding or blocking of hormone receptors [4] Major concerns about the use of BPA emerged after evidence that it could be leached out from polycarbonate drinking bottles and other commonly used plastics [5]. As a consequence, the use of BPA was reduced in several plastics, such as in baby bottles and thermal receipt papers [6,7,8]. In addition, several chemicals structurally analogous to BPA have been developed as replacements and many of them are currently in use [9]. These compounds have two hydroxyphenyl groups in their structure and are commonly referred to as BPA analogues [10]. For example, bisphenol F (4,4′-Methylenediphenol; BPF) and bisphenol S (4,4′-Sulfonyldiphenol; BPS) are two common BPA replacements used in a range of products [9,11,12]. BPF has been used in the production of lacquers, vanishers, adhesives plastics, water pipes, dental sealants, and food package coatings while BPS has been applied in the manufacturing process of polyvinyl chloride (PVC), thermal printing papers, electronic components, food and beverages containers [13,14]. Given the widespread use of these chemicals, it is not surprising that they have been detected in sediments [15], soils [16], human biofluids [17], food [18,19], wastewater treatment plants effluents [20,21], surface water [8,22], and aquatic organisms [23]. Reports of BPA being quantified in water samples started in the 1990s, with most studies being associated with river sites close to wastewater treatment plants [24]. Compared to BPA, information on the occurrence, distribution and impact on receiving organisms of BPF and BPS is still limited [9,10], which indicates the need for further examination of these chemicals that are increasingly incorporated in the production of plastic globally.

To date, the comparison of different toxicity endpoints for the three bisphenols has resulted in varying results regarding their potency in different organisms [25,26]. Acute exposure of the three bisphenols to zebrafish embryos has placed BPA as the most lethal, with a median lethal concentration (LC_50_) of 12 mg/L, followed by BPF (LC_50_ = 32 mg/L) and BPS (LC_50_ = 199 mg/L) [27]. Similarly, marine rotifer exposure to BPA, BPF, and BPS reported that BPA, with a LC_50_ value of 20.924 mg/L, is twice as lethal as BPF (LC_50_ = 42.890 mg/L) while no LC_50_ value could be determined for BPS in the concentration range tested due to the lack of lethality observed [28]. A 48-h exposure to water fleas (*Daphnia magna*) indicated that BPA, with a median effective concentration (EC_50_) of 10 mg/L, can immobilize the freshwater species at lower concentrations in comparison with BPF (EC_50_ = 56 mg/L) and BPS (EC_50_ = 55 mg/L) [29]. Interestingly, acute exposure to another water flea species (*Diaphanosoma celebensis*) revealed closer LC_50_ values to BPA (6.846 mg/L) and BPF (8.625 mg/L) in comparison with BPS (28.667 mg/L) [30]. The potential interference of BPA, BPF, and BPS with the endocrine system of different organisms has been assessed through hormone activity assays [27,29]. In a yeast assay, all three bisphenols increased estrogenic activity, with BPS exhibiting the lowest activity [29]. A study with zebrafish reported that all three bisphenols have estrogenic activity, with BPA and BPF showing similar responses [27]. Another zebrafish investigation found that BPA, BPF and BPS all act as thyroid hormone disruptors, with the analogues yielding higher potency compared to BPA [31]. In addition, a study involving human adrenal cortico-carcinoma cells observed that BPF and BPS resulted in higher steroidogenic activity than BPA [26]. Chronic exposure studies with BPA, BPF, and BPS have also yielded inconsistent responses in the same organism [32,33]. Chronic *D. magna* exposure to BPA and BPF indicated that BPF impaired reproduction at lower concentrations compared to BPA [32]. Another *D. magna* study found that only BPA and a tertiary mixture with all three bisphenols (BPA, BPF, and BPS) resulted in the inhibition of maturation [33]. Collectively, acute and chronic exposure studies suggest that BPA analogues can have similar but also unique impacts on organism function and toxicity endpoints. However, more information is required to assess how BPA analogues may alter organism health and function at the molecular-level [34]. This is particularly salient given that BPA analogue use in various products will continue to rise, and their subsequent pollution in aquatic ecosystems is likely to become more widespread.

Metabolomics, the study of low molecular-weight molecules present in cells, tissues, biofluids, is used to better understand the interactions of living organisms within their environment [35,36,37]. Metabolomic-based investigations identify responses that are directly linked to the functional state of an organism, which can be useful when studying pollutant toxicity at sub-lethal or environmentally relevant concentrations [36,38]. Although studies trying to understand how BPA alters the metabolic profile of *D. magna* [39,40,41] have reported biochemical perturbations at sub-lethal exposure concentrations, there is no information about the BPA analogues, BPF and BPS. *D. magna* is a small crustacean that occupies an intermediate level in many aquatic food chains, has a short lifespan and is highly sensitive to many pollutants [36,42]. *D. magna* has been widely used in well-established protocols for ecotoxicological studies [43,44,45] and has been listed as one of the prioritized model organisms in metabolomic-based studies [46]. Previous environmental metabolomic studies have utilized *D. magna* to understand, among others, the mode of toxic action of different pollutants [39], the impacts of chemical structure on toxicity [47], how dissolved organic matter alter the bioavailability of contaminants with varying hydrophobicity [48], and changes in lipid profiles of male and female animals with age [49].

To examine how BPA, BPF and BPS sub-lethal exposure may alter *D. magna* metabolism, this study used a targeted liquid-chromatography with tandem mass spectrometry (LC-MS/MS) method to compare metabolic responses of BPA with its analogues, BPF and BPS. This method targets polar metabolites, including amino acids, sugar derivatives, carboxylic acids, nucleosides, nucleotides, vitamins, polyamines, and neurotransmitters [50,51]. Previous studies involving *D. magna* showed that exposure to BPA elicits disruptions to amino acid and energy metabolism pathways [39,40]. Because BPA, BPF and BPS seem to induce similar physiological responses in a variety of organisms [52] and chemicals with similar structures can induce similar metabolic responses [47], we hypothesized that BPA analogues, BPF and BPS, will lead to similar patterns of metabolic disruptions to those observed to BPA. Additionally, we intend to investigate any potential correlations between exposure concentration and metabolic responses for the three bisphenols. This information will provide further insight into how metabolic profiles relate to other toxicity endpoints, such as animal immobilization and lethality.

## 2. Results

### 2.1. Multivariate Analysis with Bisphenol Exposure

**Figure 1:**
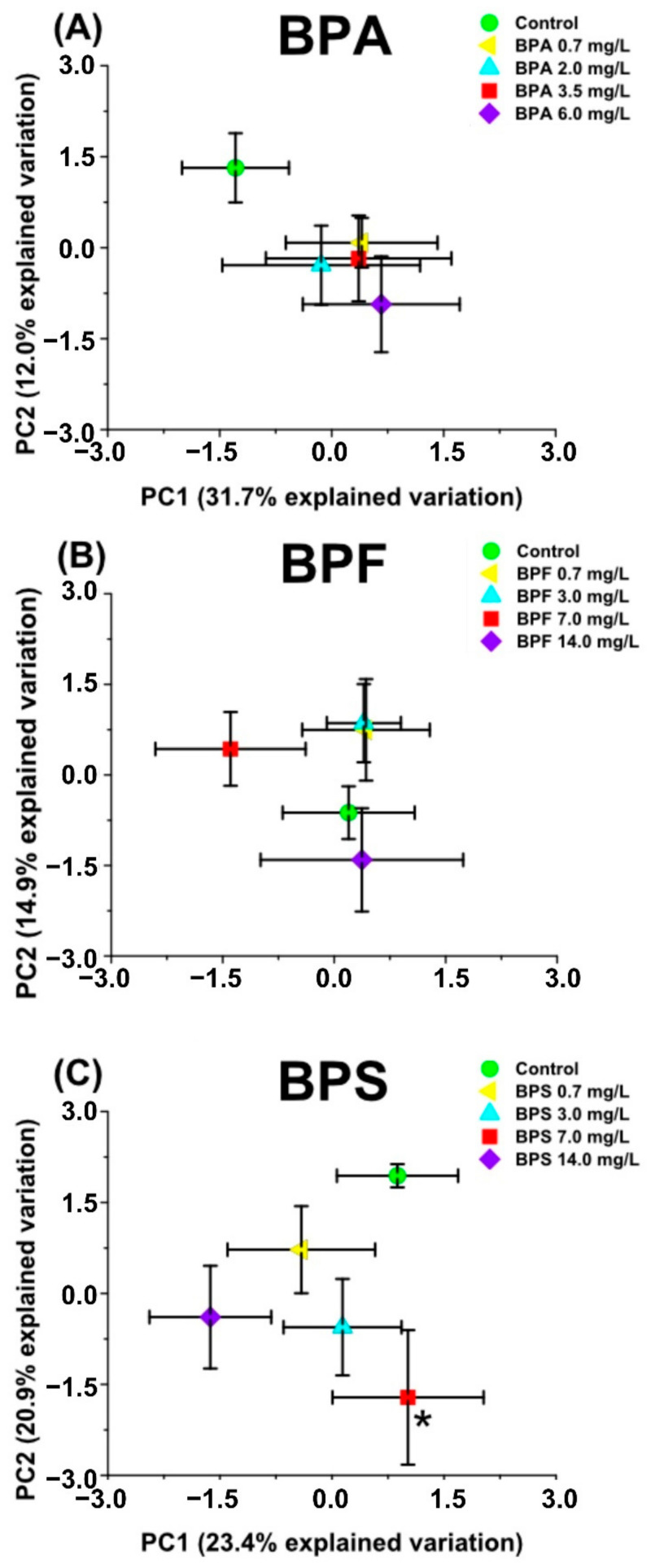
Averaged principal component analysis (PCA) score plots (PC 1 vs. PC2) of metabolic profiles of *Daphnia magna* exposed to (**A**) bisphenol A (BPA), (**B**) bisphenol F (BPF), and (**C**) bisphenol S (BPS). Averaged PCA scores are presented with their associated standard errors. Statistically significant separation from the control group (*p* ≤ 0.05) is indicated with an asterisk (*) close to the exposure group symbol.

Principal component analysis (PCA) and partial least square-discriminant analysis (PLS-DA) revealed separation between the bisphenol exposure and control groups (Figure 1 and Supplementary Materials Figures S1–S3). For both PCA and PLS-DA, components 1 and 2 explained more variation and exhibited separations that were statistically significant for at least one of the bisphenol concentrations used in the exposure. The shifts observed in the averaged PLS-DA plots, relative to the unexposed groups, were statistically significant for at least one of the exposure groups for all three bisphenols (Supplementary Materials Figures S2 and S3). Alternatively, the averaged PCA plots displayed separation (*p* ≤ 0.05) from the control group for only one BPS exposure group (Figure 1C) and one BPA exposure group (Supplementary Materials Figure S1A).

BPA exposure groups were separated from the control along principal component 2 but did not follow any concentration-dependent pattern, which is consistent with previous studies that reported non-monotonic responses with BPA exposure in different aquatic organisms [39,53,54]. Only exposure to 6 mg/L BPA was statistically significant (principal component 3; Supplementary Materials Figure S1A). Averaged PLS-DA scores plots of BPA showed that 2.0 and 6.0 mg/L exposure groups were statistically different from the control group (component 4; Supplementary Materials Figure S3A). BPF exposure did not result in significant separation from the control with PCA (Figure 1B and Supplementary Materials Figure S1B). PLS-DA displayed a statistically significant separation from the control for the lowest and the highest exposure concentrations (component 2; Supplementary Materials Figure S2B). With BPS exposure, only the 7 mg/L exposure showed separation from the control group (*p* ≤ 0.05) in the PCA plots (principal component 2; Figure 1C). After analysis via PLS-DA, the 14 mg/L was the only group statistically significantly separated from the control group (component 1; Supplementary Materials Figure S2C). Overall, neither PCA nor PLS-DA identified any concentration dependence for any of the bisphenols studied. Both multivariate analysis methods indicated that BPA and BPS exposure resulted in more distinguished separation from the control while BPF was observed to show fewer overall differences with exposure.

### 2.2. Changes in Metabolite Concentrations with Bisphenol Exposure

#### 2.2.1. Bisphenol A (BPA) Exposure

**Figure 2:**
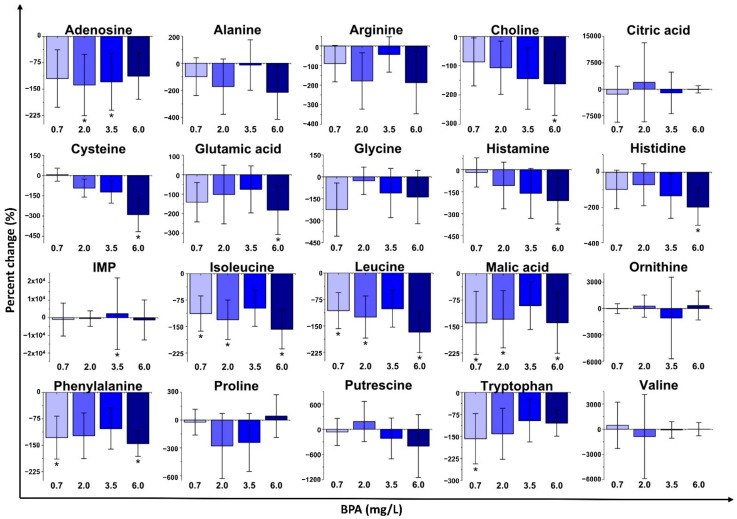
Metabolite percent changes of *D. magna* exposed to bisphenol A (BPA) at fractions of median effective concentration (EC_50_) values. The percent changes of the contaminant exposure are relative to a control. (*n* = 10 and * *p* ≤ 0.05). IMP = inosine monophosphate.

Sub-lethal exposure with BPA altered the concentration of several metabolites in *D. magna* (Figure 2 and Supplementary Materials Figure S4). The highest exposure (6.0 mg/L) accounted for most of the statistically significant changes, which is consistent with multivariate analyses (PCA and PLS-DA). BPA exposure with 6.0 mg/L downregulated the concentrations of choline, cysteine, glutamic acid, histamine, histidine, isoleucine, leucine, malic acid, and phenylalanine (*p* ≤ 0.05) relative to the control (unexposed) group. The 3.5 mg/L exposure group was less distinct from the control group. Exposure with 2.0 mg/L of BPA yielded statistically significant decreases in the concentrations of isoleucine and leucine. Acute exposure to 0.7 mg/L of BPA decreased (*p* ≤ 0.05) the concentrations of isoleucine, leucine, phenylalanine, and tryptophan. These metabolite change results, and the related biochemical roles, suggest that metabolism of branched-chain amino acids, aromatic amino acids, and protein synthesis were impacted [55,56,57]. Lastly, the metabolites with no statistically significant changes were mostly downregulated relative to the control (Supplementary Materials Figure S4). Overall, no concentration dependence was displayed for the percent changes of most individual metabolites with BPA exposure.

#### 2.2.2. Bisphenol F (BPF) Exposure

**Figure 3:**
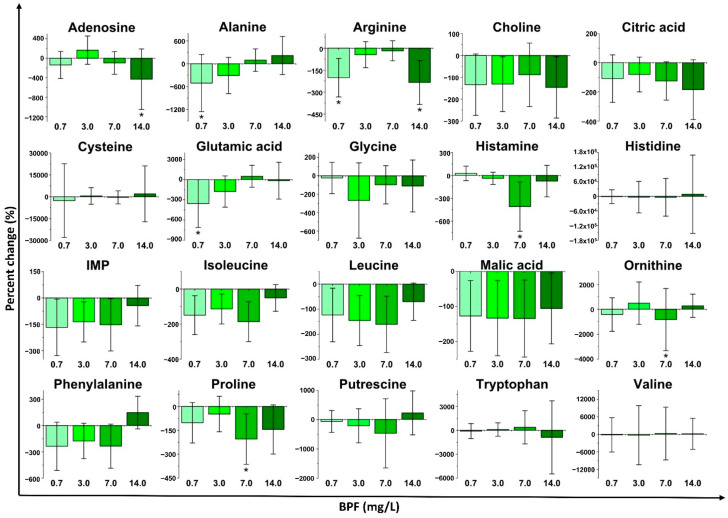
Metabolite percent changes of *D. magna* exposed to bisphenol F (BPF) at fractions of median effective concentration (EC_50_) values. The percent changes of the contaminant exposure are relative to a control. (*n* = 10 and * *p* ≤ 0.05). IMP = inosine monophosphate.

Several metabolite concentrations in *D. magna* were also altered with sub-lethal exposure to BPF (Figure 3 and Supplementary Materials Figure S5) but to a lesser extent as compared to BPA. As with the multivariate analyses, sub-lethal BPF exposure did not show any clear concentration dependence for most of the individual metabolites (Figure 3). Exposures with 0.7 and 7.0 mg/L of BPF led to the most statistically significant changes while 3.0 mg/L did not result in any statistically significantly altered metabolites. The highest BPF exposure concentration (14 mg/L) resulted in statistically significant decreases in the concentrations of arginine and adenosine. Exposure with 7.0 mg/L of BPF resulted in the downregulation of histamine, ornithine, and proline in comparison with the control group (*p* ≤ 0.05). The lowest concentration exposure (0.7 mg/L) decreased the concentration of arginine, alanine, and glutamic acid (*p* ≤ 0.05). These metabolites are associated with the metabolism of arginine, proline, glutamic acid, and protein synthesis-related pathways [58]. Similar to BPA, metabolites that did not present statistically significant changes mostly exhibited decreases in their concentration (Supplementary Materials Figure S5).

#### 2.2.3. Bisphenol S (BPS) Exposure

**Figure 4:**
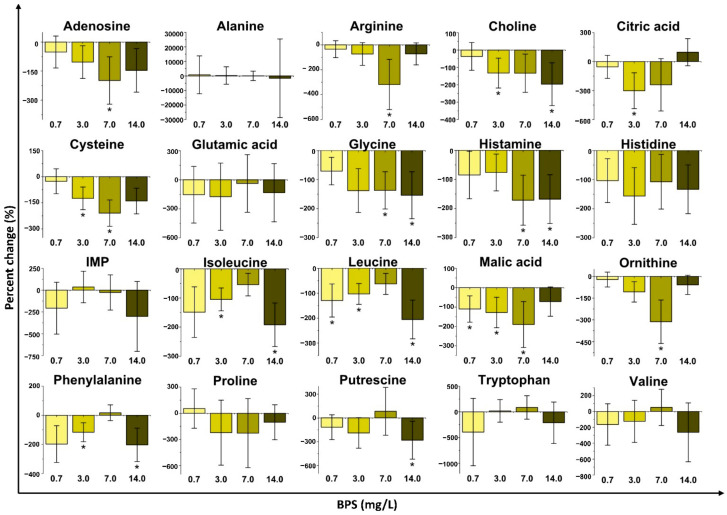
Metabolite percent changes of *D. magna* exposed to bisphenol S (BPS) at fractions of median effective concentration (EC_50_) values. The percent changes of the contaminant exposure are relative to a control. (*n* = 10 and * *p* ≤ 0.05). IMP = inosine monophosphate.

Several metabolite concentrations in *D. magna* were perturbated by the exposure to BPS (Figure 4 and Supplementary Materials Figure S6). Interestingly, a higher number of metabolites changed significantly in comparison to BPA and BPF. The three highest concentrations (3.0, 7.0 and 14 mg/L) resulted in the most changes when compared to the control group, which is consistent with PCA results (Figure 1C). The highest BPS exposure concentration (14 mg/L) significantly decreased the concentration of choline, glycine, isoleucine, leucine, phenylalanine, and putrescine. Exposure to 7.0 mg/L of BPS resulted in the downregulation of arginine, cysteine, glycine, malic acid, ornithine, and serine (*p* ≤ 0.05). After exposure to 3.0 mg/L of BPS, the concentrations of choline, citric acid, cysteine, isoleucine, leucine, malic acid, and phenylalanine significantly decreased. Finally, the lowest exposure group (0.7 mg/L) resulted in the downregulation of leucine and malic acid relative to the control group. Similar to BPA, BPS exposure groups decreased the concentration of metabolites associated with branched-chain amino acid metabolism (isoleucine, leucine, and valine) and aromatic amino acid metabolism (phenylalanine, tryptophan, and tyrosine). In a similar fashion to what was observed for BPF, metabolites linked to arginine metabolism decreased for some exposure groups. As with BPA and BPF, the metabolites that did not present statistically significant changes were mostly downregulated relative to the control group with BPS exposure (Supplementary Materials Figure S6). BPS exposure did not display concentration dependence for most metabolites.

### 2.3. Pathway Analysis and Metabolic Networks

**Figure 5:**
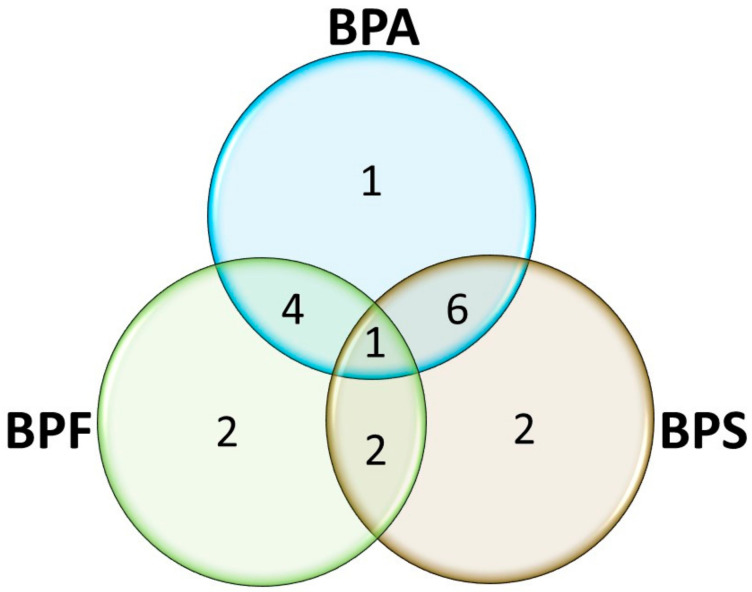
Venn diagram of pathways significantly perturbated by the different exposure groups (BPA, BPF, and BPS). The threshold for the significance level was *p* ≤ 0.05. Shared perturbated pathways: All bisphenols (1), BPA and BPS (6), BPA and BPF (4), BPF and BPS (2), unique to BPA (1), unique to BPF (2), and unique to BPS (2). Details regarding the specific pathways which were disrupted are listed in Table 1 and Supplementary Materials Table S5.

Several perturbations to biochemical pathways were identified with bisphenol exposure (Table 1 and Supplementary Materials Table S5). Overall, the different bisphenols impacted biochemical pathways similarly but also distinctly (Figure 5). BPA disturbed 12 pathways while BPS perturbated 11 pathways, and BPF a slightly smaller number of pathways (9; Table 1 and Figure 5). The aminoacyl-tRNA biosynthesis pathway was disrupted by all three bisphenols (BPA, BPF, and BPS). BPA and BPF exposures led to the perturbation of another 4 common pathways while BPA and BPS exposures shared 6 additional disturbed pathways. BPF and BPS disrupted 2 pathways in common in addition to the aminoacyl-tRNA biosynthesis pathway. BPA also disturbed 1 additional pathway while both BPF and BPF perturbated 2 additional pathways each (Figure 5).

Correlation analysis (Supplementary Materials Figures S7–S9) explored the regulation of metabolic networks amongst the measured metabolites. This type of analysis can provide information regarding the physiological state of a given metabolic system [59]. Among the correlations, those with absolute values higher than 0.5 were considered of positive correlation. BPS resulted in a higher number of metabolites with positive correlations (30) compared to BPA (19) and BPF (19). All bisphenols had similar patterns of positive correlations, including 1,3-diaminopropane/histamine, 1,3-diamiopropane/histidine, phenylalanine/isoleucine, and leucine/isoleucine (Supplementary Materials Figures S7–S9). In addition, the positive correlations of phenylalanine/guanosine/valine, alanine/methionine, malic acid/citric acid pairs were unique to BPA, BPF, and BPS, respectively, and are consistent with the results from the multivariate analyses, metabolite concentration changes and pathway analyses.

## 3. Discussion

BPA, BPF, and BPS all invoked non-monotonic responses in *D. magna* with sub-lethal exposure. Multivariate analyses (PCA and PLS-DA; Figure 1 and Supplementary Materials Figures S1–S3) as well as metabolite changes (Figure 2, Figure 3, Figure 4 and Supplementary Materials Figures S4–S6) did not reveal any concentration dependence with sub-lethal exposure to all three bisphenols studied. Non-monotonic dose/concentration responses are usually characterized by responses that do not follow commonly observed dose/concentration response patterns, where an increase of dose/concentration translates as an increase in response [60]. Interestingly, the metabolite changes observed, both with the global metabolite profile (PCA and PLS-DA) and with the individual metabolite shifts (Figure 2, Figure 3, Figure 4), also did not reflect responses that are consistent with the relative EC_50_ values reported for these pollutants. Although previous acute toxicity studies involving *D. magna* have shown that some of BPA analogues, such as BPF and BPS, have lower toxicity as defined by EC_50_ endpoints [29], our study provides evidence that these analogues can alter the *D. magna* metabolic profile to the same extent as BPA. Several other studies have also reported that sub-lethal pollutant concentrations can yield non-monotonic responses [61,62,63]. In a comparative herbicide exposure study, Jin et al. [61] observed that separated fluridone and glyphosate exposures in delta smelt altered 17β-estradiol concentrations in a non-monotonic fashion. No concentration dependence was also reported for a metabolomic-based tadpole exposure to mixtures of pharmaceuticals [63]. This aberrant behaviour of BPA and other pollutants has been suggested to be either a result of the downregulation of receptors at higher concentrations of hormone-like species or as a consequence of the integration of two or more monotonic response curves related to different pathways [4]. Collectively, these results suggest that metabolic changes with sub-lethal pollutant exposure do not linearly elicit metabolic perturbations in a manner that is consistent with other concentration-based toxicological endpoints.

To further test the hypothesis that BPA, BPF and BPS alter the metabolic profile of *D. magna* similarly, metabolite changes were used to predict disrupted biochemical pathways and revealed that the three bisphenols result in similar but also distinct biochemical perturbations (Table 1, Supplementary Materials Table S5 and Figure 5). The overall decrease displayed by most metabolites with exposure indicates that these bisphenols might act as a metabolic disruptor that results in specific metabolites to be used as a carbon source due to energetic demands [64]. Significant changes (*p* ≤ 0.05) to the concentrations of cysteine, glutamic acid, histidine, isoleucine, leucine, phenylalanine, and tryptophan for BPA, arginine, alanine, and glutamic acid for BPF, and arginine, cysteine, glycine, isoleucine, leucine, and phenylalanine for BPS are indicative of disruptions in the aminoacyl-tRNA biosynthesis (Table 1, Supplementary Materials Table S5) [65]. The aminoacyl-tRNA pathway is responsible for the pairing of amino acids with tRNA containing the corresponding anticodon prior to the protein synthesis [55]. Zebrafish exposure to BPA in a proteomic-based study also identified disruptions to aminoacyl-tRNA biosynthesis [66]. Similarly, Yue and collaborators [67] exposed human hepatoma cells to BPA and its analogues for 48 h and observed disturbances to several amino acids and energy-related metabolites that are linked to the aminoacyl-tRNA pathway. As such, it appears that all bisphenols lead to a chemical-class type metabolic response in *D. magna* as well as other environmentally relevant organisms [47,68].

In addition to a general class-based response, there is also evidence that BPA, BPF and BPS resulted in some differences with respect to metabolic pathway perturbations. For example, significant decreases (*p* ≤ 0.05) in leucine and isoleucine indicate a common disruption of branched-chain amino acid metabolism for BPA and BPS (Figure 2 and Figure 4; Table 1 and Supplementary Materials Table S5). BPA and BPS both also altered aromatic amino acid metabolism based on changes in phenylalanine concentrations with exposure (Table 1). Branched-chain amino acids (isoleucine, leucine, and valine) are mostly required for protein synthesis [56], and aromatic amino acids (tyrosine, phenylalanine, and tryptophan) are additionally involved in the synthesis of several secondary metabolites, nerve cell communication, and quenching of reactive oxygen species [57]. Acute impairment of these biochemical pathways may consequently alter protein synthesis [57,69]. Similar patterns of impairment to protein synthesis (via branched-chain amino acids and aromatic amino acid metabolism) were observed in zebrafish after exposure to BPA [70]. Our results, therefore, suggest that previously reported mechanisms for BPA exposure are also occurring with BPS sub-lethal exposure. BPA and BPF also shared perturbations to D-glutamine and D-glutamate metabolism and histamine metabolism (Table 1). Significant changes to glutamic acid (Figure 2 and Figure 3) with exposure to BPA and BPF are linked to disruptions in D-glutamine and D-glutamate metabolism. Histamine and histidine were also significantly altered and are associated with the histidine metabolism pathways that were impacted for both BPA and BPF. Exposure to BPA increased the concentration of histidine in female mussels [71] while a long-term exposure to BPA in Sprague−Dawley rats [72] also resulted in perturbations to the same pathways (D-glutamine and D-glutamate metabolism and histamine metabolism) as observed with *D. magna*. BPF resulted in the downregulation (*p* ≤ 0.05) of alanine and glutamic acid which impacts the alanine, aspartate, and glutamate metabolism, which was also reported for BPA and BPF exposure in other organisms [67,73,74]. Consequently, several common responses were observed between BPA and either BPF or BPS. However, these responses are unique to *D. magna* as not all responses observed were shared with BPA, further highlighting the need to study model organisms with analogue compounds and at different trophic levels of an ecosystem.

Interestingly, BPF and BPS also exhibited unique perturbations to the metabolome of *D. magna* that were not shared with BPA (Table 1). BPF and BPS exposures led to disruptions to arginine biosynthesis as well as arginine and proline metabolism in *D. magna* (Table 1) due to significant decreases in alanine, arginine, glutamic acid, ornithine, and proline. Arginine is involved in the synthesis of proteins, nitric oxide, creatine, polyamines, and urea and can be interconverted into the amino acids proline and glutamic acid [75]. Prenatal BPA exposure to gut bacteria in rabbits also showed perturbations to arginine-related pathways [74] such as those seen for BPF and BPS exposures with *D. magna* in our study. These findings further emphasize the importance of potential similarities between pollutants within the same chemical class. Furthermore, BPS exposure led to unique disruptions in the citric acid cycle, via the downregulation of citric acid and malic acid, and perturbations in the glycine, serine, and threonine metabolism via changes to choline, cysteine, and glycine were also observed. A study examining antibiotic impacts on a fish pathogen has shown a potential link between these two pathways, where the citric acid cycle was enhanced by glycine, serine, and threonine metabolism [76].

Overall, metabolite changes and pathway analysis demonstrated that the three bisphenols were able to impact different pathways connected to protein synthesis in both similar and distinct ways. BPA, BPF, and BPS displayed similar patterns of changes for amino acids to previous studies with *D. magna*. Nagato et al. [39] employed different BPA concentrations and reported an increase in the concentration of amino acids for exposure groups with higher concentrations of BPA and attributed this observation to a reduction in protein synthesis to counterbalance the consequences of toxic stress. In a 24-h *D. magna* exposure to BPA, Garreta-Lara et al. [41] did not observe a unique tendency of increase or decrease for the concentration of amino acids. Garreta-Lara et al. [41] also reported a downregulation in the biosynthesis of fatty acids and suggested a major disruption to sugar and lipid metabolism was responsible for the metabolic responses, rather than a response to toxic stress. This disturbance may result in amino acids being used as a carbon source for energic requirements, which agrees with our observations for all three bisphenols. Furthermore, according to the KEGG database [77,78], the pathway that was impacted by all bisphenols (aminoacyl-tRNA biosynthesis) is related to those commonly perturbated by BPA and BPF (histidine metabolism), BPA and BPS (phenylalanine, tyrosine, and tryptophan biosynthesis; valine, leucine, and isoleucine biosynthesis; and glycine, serine, and threonine metabolism), and BPF and BPS (arginine and proline metabolism). Pathways such as glycine, serine, and threonine metabolism, phenylalanine, tyrosine, and tryptophan biosynthesis, and arginine and proline metabolism are related to other energy-related pathways such as glycolysis/gluconeogenesis, citric acid cycle, and pentose phosphate pathways [77,78]. These results further emphasize that BPA exposure leads to energetic impairments in *D. magna* [39] and is also likely the main mode of action of BPF and BPS exposure. This further supports our findings that bisphenols impart a chemical-class response in acute sub-lethal exposure but also exhibit some exclusive metabolic differences dependent on the specific concentration and type of bisphenol.

## 4. Materials and Methods

### 4.1. Daphnia magna Culturing

The *D. magna* culture was originally purchased from Ward Science (St. Catherines, ON, Canada) and has been maintained since 2013 based on guidelines from the Ontario Ministry of the Environment [79]. The culture was kept in dechlorinated tap water (hardness of around 127 mg CaCO_3_ mg/L), at temperatures of roughly 20 °C, and under a light/dark cycle of 16/8 h. The daphnids were fed consistently with an algae mixture of *Chorella vulgaris* and *Rhaphodocelis subcapitata*. The algae were also cultured based on the guidelines from the Ontario Ministry of the Environment [80]. Twice a week, approximately 20% of the *Daphnia* culture media was replaced with fresh media to maintain consistent water quality. Selenium, in the form of sodium selenite pentahydrate (≥98% purity, Sigma-Aldrich, Mississauga, ON, Canada) and vitamin B12 (cyanocobalamin; ≥95.0% purity, TCI America, Portland, OR, USA) were added to the media as supplements twice a week, each at a concentration of 1 μg/L [79].

### 4.2. Sub-Lethal Bisphenol Exposure

Adult daphnids (approximately 14 days old) were exposed for 48 h to sub-lethal concentrations of BPA (≥99% purity Sigma-Aldrich, Mississauga, ON, Canada), BPS (≥98% purity, Sigma-Aldrich, Mississauga, ON, Canada), or BPF (≥98% purity, Sigma-Aldrich, Mississauga, ON, Canada). The sub-lethal concentrations used in this study were based on the EC_50_ values reported in the literature (Supplementary Materials Table S1) [29]. Preliminary tests were performed to confirm the survival of *D. magna* at the highest sub-lethal concentration for each of the bisphenols. The 48-h exposure was carried out with each adult daphnid placed in a 20 mL scintillation vial containing 15 mL of water. The exposure concentrations used included: 0.7, 2.0, 3.5, 6.0 mg/L for BPA, and 0.7, 3.0, 7.0, 14 mg/L for each of BPF and BPS. The control (unexposed) group used the same procedure as the exposed groups but did not include any bisphenols. Each exposure concentration (or unexposed control) was replicated 10 times. All concentrations of BPA, BPF, and BPS, as well as control groups, were analyzed by high-performance liquid chromatography with diode-array detector (HPLC-DAD; Supplementary Materials) [81]. Measured bisphenol concentrations were consistent with nominal concentrations (Supplementary Materials Table S2) with some small losses observed, likely due to binding to algae [82]. The daphnids were fed with the same algae mixture 24 h after the start of the exposure. After the exposure, each daphnid was transferred into a 1.5 mL microcentrifuge tube, rinsed with deionized water (~18 MΩ/cm; Millipore Synergy UV system, Molsheim, France), and flash-frozen with liquid nitrogen. The daphnids were then freeze-dried for 48 h and stored at −20 °C until the metabolite extraction.

### 4.3. Metabolite Extraction and Liquid Chromatography-Tandem Mass Spectrometry (LC-MS/MS) Analysis

The polar metabolites were extracted according to the method of Jeong and Simpson [51]. All extractions were carried out with samples placed on ice to ensure metabolite preservation. Briefly, 160 µL of a mixture of methanol (≥99.9% purity, Fisher Chemical, Fair Lawn, NJ, USA)/deionized water (~18 MΩ/cm; Millipore Synergy UV system, Molsheim, France) (10:9, *v/v*) was added to each lyophilized daphnid in a 1.5 mL microcentrifuge tube. The daphnid and solvent mixture were homogenized with a motorized pestle. After that, 84.5 µL of a mixture of internal standards were added to monitor extraction efficiency: acyclovir (98% purity, Sigma-Aldrich, Mississauga, ON, Canada), glycine-d_2_ (98% purity, Cambridge Isotope Laboratories, Tewksbury, MA, USA), and phenyl-d_5_-alanine (98% purity, Sigma-Aldrich, Mississauga, ON, Canada). Following, 600 µL of the methanol/water mixture was added to each tube. The microcentrifuge tubes were then sonicated for 5 min followed by the addition of 400 µL of chloroform (99.5%, Alfa Aesar, Ottawa, ON, Canada). The samples were then manually mixed for 1 min and then centrifuged (12,500 rpm, 5 min, 4 °C) to separate the methanol/water phase from the chloroform. This part of the extraction cycle was repeated twice for a total of three extractions and all extracts were combined. 200 µL of the combined supernatants were isolated and filtered with a 0.2 µm syringeless filter (GE Healthcare, Buckinghamshire, UK) before the LC-MS/MS analysis.

Metabolites were quantified using an Agilent 1260 LC system coupled with a 6420A triple quadrupole MS. An Ultra AQ C_18_ chromatographic column (100 mm × 4.6 mm × 3 µm; Restek, Center County, PA, USA) was used to separate the metabolites. The mobile phases included: deionized water (~18 MΩ/cm; Millipore Synergy UV system, Molsheim, France) and acetonitrile (≥99.9% purity, Fisher Chemical, Fair Lawn, NJ, USA), both with 0.1% of formic acid (LC/MS grade, Fisher Chemical, Geel, Belgium) as a modifier. Electrospray Ionization was applied in both positive and negative modes and ions were quantified using Multiple Reaction Monitoring (Supplementary Materials Table S4). The three internal standards (acyclovir, glycine-d_2_ and phenyl-d_5_-alanine) were also added to account for ionization efficiency of the metabolites in the samples and possible matrix effects. Each metabolite had an assigned internal standard (Supplementary Materials Table S4) and ratios of the metabolite peak area to the internal standard peak area were calculated to normalize the area counts. The area ratios were then used in the generation of external calibration curves. Stock solutions of metabolites were prepared with analytical standards (≥98% purity; Supplementary Materials Table S4) in a mixture water/methanol (1:1, *v/v*). The standard solutions were obtained by a serial dilution with the water/methanol mixture. The employed method can detect up to 51 metabolites [51]. In this study, 42 metabolites in *D. magna* were detected above the limit of quantification. Agilent Mass Hunter Quantitative Analysis program (B.08.00) was employed in the process of peak integration of metabolites and internal standards as well as in process of signal-to-noise ratio estimation. Finally, the generation of external standard calibration curves and the calculation of sample concentration were performed using Microsoft Excel (Microsoft Office 365, Redmond, WA, USA).

### 4.4. Data Processing and Pathway Interpretation

The calculated concentrations were inputted into MetaboAnalystR 5.0 (https://www.metaboanalyst.ca/, accessed on 4 of July of 2021) and filtered to remove outliers and near-constant values [83,84]. Outliers and near-constant values were estimated using the interquartile range (IQR). The values that were identified as below the limit of quantification were replaced with 1/5 of the minimum positive values in the original data for a specific metabolite. Each metabolite concentration was then normalized by the sum of the concentrations of all metabolites for each sample using MetaboAnalystR. Lastly, the normalized concentration data were scaled using the tool Autoscaling, present in MetaboAnalystR.

Statistical analyses were performed using MetaboAnalystR package [83,84]. PCA and PLS-DA matrixes of scores were obtained. The data were imported into Microsoft Excel and averaged score plots were generated. Figures for the averaged score plots were created using OriginPro 8 (OriginLab, Northampton, MA, USA). The separation between exposure and control groups on averaged PCA and PLS-DA scores plots was assessed using one-way analysis of variance (ANOVA) followed by a post-doc test, Tukey’s range test. A *p*-value of ≤0.05 was used to assess the significance level. Individual metabolite percent changes of each exposure group were calculated by subtracting the normalized concentration of a given metabolite in an exposure group from the normalized concentration of the same metabolite in the control, then dividing this difference by the normalized concentration of the metabolite in the control. A *t*-test (two-tailed, equal variance, α ≤ 0.05) was then used to identify the statistically significant differences between metabolites from exposure and control groups.

Pathway analysis was performed using the MetaboAnalystR package [83,84]. A list of all metabolites that presented statistically significant changes relative to the control was inputted into the web-based tool. The KEGG pathway library for *Drosophila melanogaster* was selected to be used as the reference organism due to their evolutionary proximity [85]. Pathways that matched with a *p*-value of ≤0.05 were considered statistically significant.

## 5. Conclusions

This study investigated how the metabolic profile of *D. magna* changed upon exposure to BPA and its analogues, BPF and BPS, at sub-lethal concentrations. The *D. magna* metabolite changes revealed that the three bisphenols can elicit similar and dissimilar responses. Changes in metabolites associated with amino acid-related pathways point toward protein synthesis impairment. The downregulation observed for most metabolites in exposure to BPA, BPF, BPS suggests that some metabolites, especially amino acids, might be used as carbon sources to cope with energetic needs. Non-monotonic responses were common to all three bisphenols and are consistent with previous reports on the exposure of BPA to *D. magna*. Interestingly, we did not find any correlations between effective concentration (EC_50_) and metabolic responses, indicating the need for assessments using different endpoints, especially for chemicals within the same class. Further studies addressing other bisphenol analogues might shed more light on perturbations observed, confirm the suggested impairments on protein synthesis and determine if this is a ubiquitous chemical class impairment. Additionally, our study demonstrates the importance of incorporating metabolomic investigations in exposure assessment for aquatic ecosystems.

## Figures and Tables

**Table 1:** MetaboAnalyst evaluation for major biochemical pathways impacted by bisphenol exposure (*p* ≤ 0.05). Pathway in **red**: common to all three bisphenols (1). Pathways in **blue**: common between BPA and BPS (6). Pathways in **green**: common between BPA and BPF (4). Pathways in **purple**: common between BPF and BPS (2). Pathways in **black**—unique pathways.

**Pathways**	**Compounds**	**Metabolites Associated**
**Aminoacyl-tRNA biosynthesis**	BPA, BPF, and BPS	Alanine, Arginine, Cysteine, Glutamic acid, Glycine, Histidine, Isoleucine, Leucine, Phenylalanine, Tryptophan
**Valine, leucine, and isoleucine biosynthesis**	BPA and BPS	Leucine, Isoleucine
**Valine, leucine, and isoleucine degradation**	BPA and BPS	Leucine, Isoleucine
**Phenylalanine, tyrosine, and tryptophan biosynthesis**	BPA and BPS	Phenylalanine
**Glyoxylate and dicarboxylate metabolism**	BPA and BPS	Citric acid, Malic acid, Glutamic acid
**Glutathione metabolism**	BPA and BPS	Cysteine, Glutamic acid, Glycine, Ornithine, Putrescine
**Glycine, serine, and threonine metabolism**	BPA and BPS	Choline, Cysteine, Glycine
**Purine metabolism**	BPA and BPF	Adenosine, Inosine monophosphate
**Histidine metabolism**	BPA and BPF	Histidine, Histamine
**Nitrogen metabolism**	BPA and BPF	Glutamic acid
**D-Glutamine and D-glutamate metabolism**	BPA and BPF	Glutamic acid
**Arginine biosynthesis**	BPF and BPS	Arginine, Glutamic acid, Ornithine
**Arginine and proline metabolism**	BPF and BPS	Alanine, Arginine, Glutamic acid, Proline, Ornithine
**Phenylalanine metabolism**	BPA	Phenylalanine
**Alanine, aspartate, and glutamate metabolism**	BPF	Alanine, Glutamic acid
**Butanoate metabolism**	BPF	Glutamic acid
**Citrate cycle (TCA cycle)**	BPS	Citric acid, Malic acid
**Pyruvate metabolism**	BPS	Malic acid

## Data Availability

The data presented in this study are available in the article itself and in the Supplementary Materials.

## Supplementary Materials

The following are available online at https://www.mdpi.com/article/10.3390/metabo11100666/s1: Table S1: Relevant physical properties and toxicity to Daphnia magna of bisphenol A (BPA), bisphenol S (BPS), and bisphenol F (BPF), Table S2: Exposure concentrations and standard deviations (SD) for BPA, BPF, BPS, and control measured using high-performance liquid chromatography with a diode array detector (HPLC-DAD), Table S3: Liquid chromatography-tandem mass spectrometry (LC-MS/MS) parameters used for electrospray ionization (ESI), Table S4: Metabolite class, purity of standards retention time, MRM, ESI Polarity, and internal standard used for the LC-MS/MS analysis, Table S5: MetaboAnalyst evaluation for major biochemical pathways impacted by bisphenol exposure. Pathway in red: common to all three bisphenols (1). Pathways in blue: common between BPA and BPS (6). Pathways in green: common between BPA and BPF (4). Pathways in purple: common between BPF and BPS (2). Pathways in black—unique pathways, Figure S1: Averaged principal component analysis (PCA) score plots (PC 1 vs. PC2) of metabolic profiles of D. magna exposed to (A) bisphenol A (BPA), (B) bisphenol B (BPF), and (C) bisphenol S (BPS). Averaged PCA scores are presented with their associated standard error. Statistically significant separation from the control group (*p* ≤ 0.05) is indicated with an asterisk (*) close to the exposure group symbol, Figure S2: Averaged partial least square-discriminant analysis (PLS-DA) score plots (Comp. 1 vs. Comp. 3) of metabolic profiles of D. magna exposed to (A) bisphenol A (BPA), (B) bisphenol B (BPF), and (C) bisphenol S (BPS). Averaged PCA scores are presented with their associated standard error. Statistically significant separation from the control group (*p* ≤ 0.05) is indicated with an asterisk (*) close to the exposure group symbol, Figure S3: Averaged partial least square-discriminant analysis (PLS-DA) score plots (Comp. 3 vs. Comp. 4) of metabolic profiles of D. magna exposed to (A) bisphenol A (BPA), (B) bisphenol B (BPF), and (C) bisphenol S (BPS). Averaged PCA scores are presented with their associated standard error. Statistically significant separation from the control group (*p* ≤ 0.05) is indicated with an asterisk (*) close to the exposure group symbol, Figure S4: Metabolite percent changes of D. magna exposed to bisphenol A (BPA) at fractions of EC50 values. The nominal concentrations were 0.7, 2.0, 3.5, and 6.0 mg/L. The percent changes of the contaminant exposure are relative to a control. (n = 10 and * *p* ≤ 0.05). 1,3-DAP = 1,3-diaminopropane, AMP = adenosine monophosphate, G-6-P = glucose-6-monophosphate, GMP = guanosine monophosphate, IMP = inosine monophosphate, Figure S5: Metabolite percent changes of D. magna exposed to bisphenol F (BPF) at fractions of EC50 values. The nominal concentrations were 0.7, 3.0, 7.0, and 14.0 mg/L. The percent changes of the contaminant exposure are relative to a control. (n = 10 and * p ≤ 0.05). 1,3-DAP = 1,3-diaminopropane, AMP = adenosine monophosphate, G-6-P = glucose-6-monophosphate, GMP = guanosine monophosphate, IMP = inosine monophosphate, Figure S6: Metabolite percent changes of D. magna exposed to bisphenol S (BPS) at fractions of EC50 values. The nominal concentrations were 0.7, 3.0, 7.0, and 14.0 mg/L. The percent changes of the contaminant exposure are relative to a control. (n = 10 and * *p* ≤ 0.05). 1,3-DAP = 1,3-diaminopropane, AMP = adenosine monophosphate, G-6-P = glucose-6-monophosphate, GMP = guanosine monophosphate, IMP = inosine monophosphate, Figure S7: Pearson correlation heatmap for bisphenol A (BPA) exposure which demonstrates the correlation between metabolites. Dark red indicates a strong positive correlation and dark blue represents a strong negative correlation between the two given metabolites. 1,3-DAP = 1,3-diaminopropane, AMP = adenosine monophosphate, G-6-P = glucose-6-monophosphate, GMP = guanosine monophosphate, IMP = inosine monophosphate, Figure S8: Pearson correlation heatmap for bisphenol F (BPF) exposure which demonstrates the correlation between metabolites. Dark red indicates a strong positive correlation and dark blue represents a strong negative correlation between the two given metabolites. 1,3-DAP = 1,3-diaminopropane, AMP = adenosine monophosphate, G-6-P = glucose-6-monophosphate, GMP = guanosine monophosphate, IMP = inosine monophosphate, Figure S9: Pearson correlation heatmap for bisphenol S (BPS) exposure which demonstrates the correlation between metabolites. Dark red indicates a strong positive correlation and dark blue represents a strong negative correlation between the two given metabolites. 1,3-DAP = 1,3-diaminopropane, AMP = adenosine monophosphate, G-6-P = glucose-6-monophosphate, GMP = guanosine monophosphate, IMP = inosine monophosphate.
