# Supplementary material for: Metabolomics Reveals That Bisphenol Pollutants Impair Protein Synthesis-Related Pathways in Daphnia magna
Source: Metabolites. 2021 Sep 29;11(10):666. doi: 10.3390/metabo11100666 (PMC8540811; doi:10.3390/metabo11100666)
Supplement: Supplementary file 1 [file metabolites-11-00666-s001.zip › BP SI revised clean version.pdf]

Supplementary Materials

Metabolomics reveals that bisphenol pollutants impair protein synthesis-related pathways in *Daphnia magna*

Erico A. Oliveira Pereira [1], Lisa M. Labine [1,2], Sonya Kleywegt [3], Karl J. Jobst [4], André J. Simpson [1,2], and Myrna J. Simpson [1,2]*

[1] Environmental NMR Centre and Department of Physical and Environmental Sciences, University of Toronto Scarborough, 1265 Military Trail, Toronto, ON, M1C 1A4, Canada; erico.pereira@mail.utoronto.ca
[2] Department of Chemistry, University of Toronto, 80 St. George St., Toronto, ON, M5S 3H6, Canada; lisa.labine@mail.utoronto.ca; andre.simpson@utoronto.ca
[3] Technical Assessment and Standards Development Branch, Ontario Ministry of the Environment, Conservation and Parks, Toronto, ON, M4V 1M2, Canada; sonya.kleywegt@ontario.ca
[4] Department of Chemistry, Memorial University of Newfoundland, St. John's, NL, A1B 3X7, Canada; karl.jobst@mun.ca
* Correspondence: myrna.simpson@utoronto.ca; Tel.: + 1-416-287-7234

Table S1: Relevant physicochemical properties and lethality and immobilization tests for *Daphnia magna* of bisphenol A (BPA), bisphenol S (BPS), and bisphenol F (BPF).

**Bisphenol structure**	**Aqueous solubility [1]**	**Henry's law constant [2]**	**Log octanol-water partition coefficient (log K_{OW}) [3]**	**Lethality and immobilization tests for *D. magna***
**Bisphenol A (BPA)** 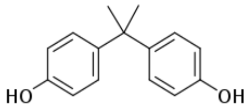	120 - 300 mg/L	4.0×10^{-11} atm m^3/mol at 25 °C	3.64	LC_{50} (48-h) = 14.4 mg/L [1]<br><br>EC_{50} (48-h) = 10 mg/L [2]
**Bisphenol S (BPS)** 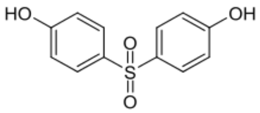	1100 mg/L	2.7×10^{-15} atm m^3/mol at 25 °C	1.65	EC_{50} (48-h) = 55 mg/L [2]
**Bisphenol F (BPF)** 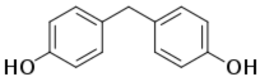	190 mg/L	5.2×10^{-12} atm m^3/mol at 25 °C	3.06	EC_{50} (48-h) = 56 mg/L [2]

[1] Chemspider (http://www.chemspider.com/) [2] PubChem (https://pubchem.ncbi.nlm.nih.gov/) [3] [3]

Quantification of Bisphenol A (BPA), Bisphenol F (BPF), and Bisphenol S (BPS) in Exposure Water

The nominal concentrations of bisphenol A (BPA), bisphenol F (BPF), and bisphenol S (BPS) before and after exposure were confirmed using high-performance liquid chromatography with a diode array detector (HPLC-DAD). Aliquots from 3 randomly chosen replicates were collected at 0 and 48 h. The samples were passed through a 0.2 μm PTFE syringe filter (Thermo Scientific, Rockwood, TN, USA) before the chromatographic analysis. The separation of the 3 pollutants was carried out with an Agilent LC (1100 Series) using the method of Rezaee et al. [4]. A reverse-phase C_{18} column (250 mm × 4.6 mm × 5 μm; Grace Discovery Sciences, Columbia, MD) was used and the mobile phases employed were deionized water (~18 MΩ/cm; Millipore Synergy UV system, Molsheim, France) and acetonitrile (≥99.9% purity, Fisher Chemical, Fair Lawn, NJ, USA) (55:45, v/v). A flow rate of 1 mL/min in an isocratic elution mode was utilized. The injection volume was 20 μL for all samples and the wavelength used for the detection was 224 nm. The

results of the concentration confirmation for the three pollutants (BPA, BPF, and BPS) are listed in Table S2. Some exposure groups showed small decreases in their concentration over the 48 h-period, but they were still within the 95% confidence interval. The only exceptions were the exposure groups with BPA 2.0 and 3.5 mg/L.

Table S2: Exposure concentrations and standard deviations (SD) for bisphenol A, bisphenol F, bisphenol S, and control groups measured using high-performance liquid chromatography with a diode array detector (HPLC-DAD).

		Start of the exposure (0 h)		After the exposure (48 h)	
	**Nominal concentration (mg/L)**	**Measured concentrations ± SD (mg/L)**	**Confidence interval (95%) (mg/L)**	**Measured concentrations ± SD (mg/L)**	**Confidence interval (95%) (mg/L)**
**Bisphenol A (BPA)**	0.7	0.75 ± 0.01	0.75 ± 0.02	0.62 ± 0.04	0.62 ± 0.11
	2.0	1.69 ± 0.08	1.69 ± 0.21	1.56 ± 0.14	1.56 ± 0.36
	3.5	2.74 ± 0.20	2.74 ± 0.51	2.65 ± 0.17	2.65 ± 0.43
	6.0	5.71 ± 0.42	5.71 ± 1.05	5.47 ± 0.50	5.47 ± 1.25
**Bisphenol F (BPF)**	0.7	0.67 ± 0.01	0.67 ± 0.03	0.62 ± 0.01	0.62 ± 0.02
	3.0	2.98 ± 0.08	2.98 ± 0.19	2.80 ± 0.11	2.80 ± 0.27
	7.0	6.51 ± 0.11	6.51 ± 0.27	6.09 ± 0.26	6.09 ± 0.66
	14.0	13.45 ± 0.13	13.45 ± 0.33	12.93 ± 0.37	12.93 ± 0.91
**Bisphenol S (BPS)**	0.7	0.61 ± 0.03	0.61 ± 0.06	0.64 ± 0.02	0.64 ± 0.04
	3.0	2.99 ± 0.03	2.99 ± 0.09	2.83 ± 0.04	2.83 ± 0.10
	7.0	6.89 ± 0.09	6.89 ± 0.24	6.79 ± 0.06	6.79 ± 0.15
	14.0	14.04 ± 0.08	14.04 ± 0.21	13.7 ± 0.10	13.7 ± 0.25
**Control**	-	Below detection limits [1]	-	Below detection limits [1]	-

[1] Limits of detection: BPA (0.02 mg/L), BPF (0.08 mg/L) and BPS (0.04 mg/L)

Metabolite Analysis via Liquid Chromatography-Tandem Mass Spectrometry (LC-MS/MS)

Additional parameters regarding the ionization process for the LC-MS/MS analysis are listed in Table S3. Mobile phases used were deionized water (~18 MΩ/cm; Millipore Synergy UV system, Molsheim, France) and acetonitrile (≥99.9% purity, Fisher Chemical, Fair Lawn, NJ, USA), both with

0.1% of formic acid (LC/MS grade, Fisher Chemical, Geel, Belgium) added as a modifier. The chromatographic separation was carried out using a gradient of 5% of acetonitrile at time zero, 40%, 60%, 80% and 5% of the same solvent at 3.75, 4.50, 6.75, 8.50 min, respectively. The gradient was followed by a 5-min equilibration period. A flow rate of 1 mL/min was used with an injection volume of 10 μL. All calibration curves had at least 5 consecutive concentration levels. Blanks were run in between every 10 samples to investigate sample carryover and standards checks were run at the beginning and at the end of the analysis. The coefficient of determination (R^2) for all metabolite calibration curves was above 0.98. Information regarding metabolite class, retention time, Multiple Reaction Monitoring (MRM), Electrospray Ionization (ESI) polarity, and internal standard are listed in Table S4.

Table S3: Liquid chromatography-tandem mass spectrometry (LC-MS/MS) parameters used for electrospray ionization (ESI).

Parameters	Details
**Temperature (drying gas)**	300°C
**Flow rate (drying gas)**	6 L/min
**Pressure (nebulizer)**	15 psi
**Capillary voltage**	4000 V

Table S4: Metabolite class, purity of standards retention time, Multiple Reaction Monitoring (MRM), ESI Polarity, and internal standard used for the LC-MS/MS analysis

Metabolite (Purity)	Class	Retention time (min)	MRM (m/z)	ESI polarity	Internal standard
**1,3-Diaminopropane (≥99%)**	Polyamine	0.885	75.0 -> 58.2	Positive	Glycine-d_2
**Putrescine (≥98%)**	Amino acid derivative	0.893	89.0 -> 72.0	Positive	Glycine-d_2
**Histamine (≥98%)**	Neurotransmitter	0.911	112.0 -> 95.1	Positive	Glycine-d_2
**Ornithine (≥98%)**	Amino acid	0.966	133.0 -> 70.0	Positive	Glycine-d_2
**Histidine (≥98.5%)**	Amino acid	1.021	156.1 -> 110.1	Positive	Glycine-d_2
**Arginine (≥98.5%)**	Amino acid	1.030	175.1 -> 70.2	Positive	Glycine-d_2
**Cysteine (≥99%)**	Amino acid derivative	1.048	241.0 -> 74.1	Positive	Glycine-d_2

**Glycine-d₂ (ITSD) (≥98%)**	Deuterated amino acid	1.063	78.1 -> 32.2	Positive	-
**Glycine (≥99%)**	Amino acid	1.064	76.0 -> 30.2	Positive	Glycine-d_2
**Serine (≥99%)**	Amino acid	1.072	106.1 -> 60.2	Positive	Glycine-d_2
**S-adenosyl methionine (≥98%)**	Amino acid derivative	1.085	399.0 -> 250.0	Positive	Glycine-d_2
**Glutamine (≥98.5%)**	Amino acid	1.107	147.1 -> 84.1	Positive	Glycine-d_2
**Threonine (≥98.5%)**	Amino acid	1.109	120.1 -> 74.2	Positive	Glycine-d_2
**Homoserine (≥99%)**	Amino acid derivative	1.109	120.0 -> 56.0	Positive	Glycine-d_2
**Alanine (≥98.5%)**	Amino acid	1.110	89.9 -> 44.2	Positive	Glycine-d_2
**GABA (≥99%)**	Neurotransmitter	1.119	104.1 -> 87.1	Positive	Glycine-d_2
**Glutamic acid (≥99%)**	Amino acid	1.125	148.1 -> 84.1	Positive	Glycine-d_2
**Thiamine (≥99%)**	Vitamin	1.142	265.0 -> 122.0	Positive	Glycine-d_2
**Citrulline (≥98%)**	Amino acid	1.153	176.0 -> 70.0	Positive	Glycine-d_2
**Glucose-6-phosphate (≥98%)**	Monosaccharide derivative	1.159	259.0 -> 78.6	Negative	Glycine-d_2
**Aspartic acid (≥98%)**	Amino acid	1.212	134.1 -> 74.1	Positive	Glycine-d_2
**Cytidine monophosphate (≥98%)**	Nucleotide	1.218	324.0 -> 112.0	Positive	Acyclovir
**Acetyllysine (≥99%)**	Amino acid derivative	1.247	189.2 -> 84.2	Positive	Glycine-d_2
**Carnitine (≥98%)**	Amino acid derivative	1.247	162.0 -> 103.0	Positive	Glycine-d_2
**Choline (≥98%)**	Neurotransmitter	1.261	104.0 -> 60.0	Positive	Glycine-d_2
**Proline (≥99%)**	Amino acid	1.298	116.1 -> 70.2	Positive	Glycine-d_2
**Malic acid (≥99%)**	Carboxylic acid	1.329	133.0 -> 114.8	Negative	Glycine-d_2

**Adenosine monophosphate (≥98%)**	Nucleotide	1.331	348.0 -> 136.0	Positive	Acyclovir
**Valine (≥99%)**	Amino acid	1.336	118.1 -> 72.0	Positive	Glycine-d_2
**Citric acid (≥99%)**	Carboxylic acid	1.348	191.0 -> 111.0	Negative	Glycine-d_2
**Uridine monophosphate (≥98%)**	Nucleotide	1.652	325.0 -> 97.0	Positive	Acyclovir
**Methionine (≥98.5%)**	Amino acid	1.843	150.1 -> 104.1	Positive	Glycine-d_2
**Guanosine monophosphate (≥99%)**	Nucleotide	1.850	364.0 -> 152.0	Positive	Acyclovir
**Inosine monophosphate (≥98%)**	Nucleotide	1.850	349.0 -> 137.0	Positive	Acyclovir
**Dopamine (≥98%)**	Neurotransmitter	1.852	154.1 -> 137.0	Positive	Glycine-d_2
**Nicotinic acid (≥99%)**	Vitamin	1.864	124.1 -> 80.3	Positive	Glycine-d_2
**Acetylcholine (≥98%)**	Neurotransmitter	1.882	146.1 -> 87.0	Positive	Glycine-d_2
**Uridine (≥99.5%)**	Nucleoside	2.012	245.0 -> 113.0	Positive	Acyclovir
**Isoleucine (≥98.5%)**	Amino acid	2.138	132.1 -> 86.2	Positive	Glycine-d_2
**Leucine (≥99%)**	Amino acid	2.138	132.1 -> 86.2	Positive	Glycine-d_2
**Tyrosine (≥99%)**	Amino acid	2.258	182.1 -> 165.0	Positive	Phenyl-d_5-alanine
**Tyramine (≥98%)**	Amino acid	2.336	138.0 -> 121.0	Positive	Phenyl-d_5-alanine
**Adenosine (≥99%)**	Nucleoside	2.663	268.0 -> 136.0	Positive	Acyclovir
**Inosine (≥99%)**	Nucleoside	2.823	269.0 -> 137.0	Positive	Acyclovir
**Guanosine (≥99%)**	Nucleoside	2.879	284.0 -> 152.2	Positive	Acyclovir
**Acyclovir (ITSD) (≥98%)**	Nucleoside analogue	2.974	226.1 -> 151.9	Positive	-
**Phenyl-d_5-alanine (ITSD) (≥98%)**	Deuterated amino acid	3.257	171.1 -> 106.1	Positive	-

**Phenylalanine (≥99%)**	Amino acid	3.286	166.1 -> 120.1	Positive	Phenyl-d_5-alanine
**Tryptophan (≥98%)**	Amino acid	4.058	205.1 -> 188.1	Positive	Phenyl-d_5-alanine
**Riboflavin (≥98%)**	Vitamin	4.626	377.0 -> 243.0	Positive	Phenyl-d_5-alanine

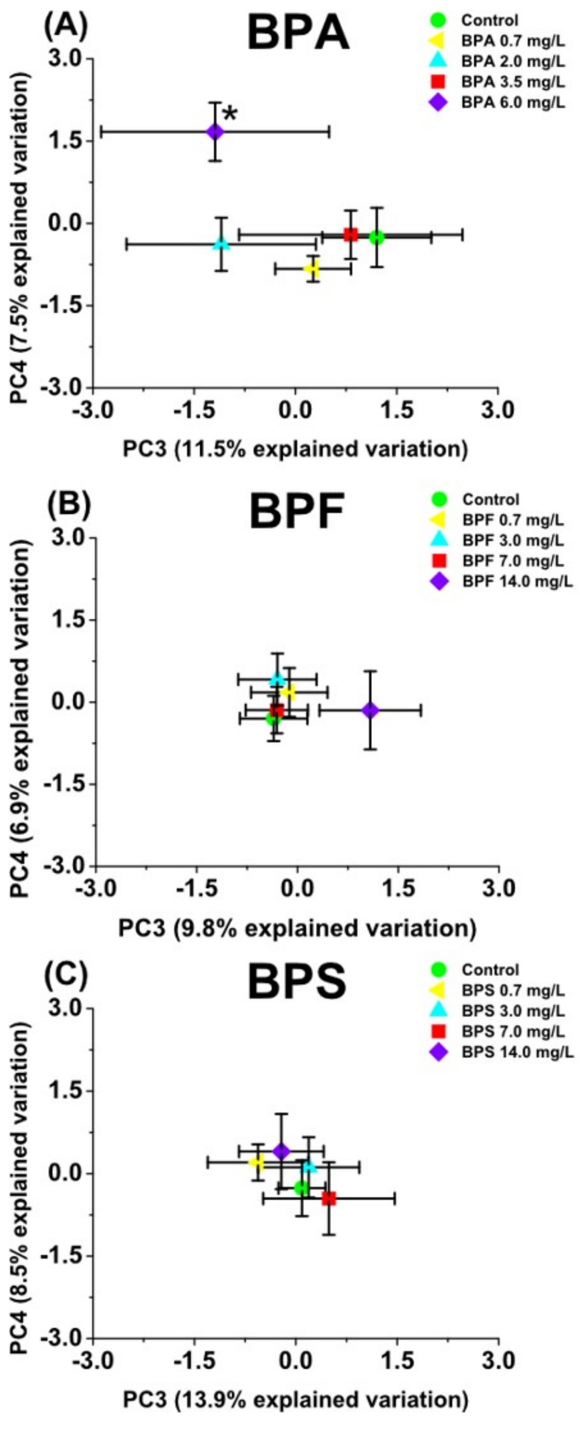

Figure S1: Averaged principal component analysis (PCA) score plots (PC 1 vs. PC2) of metabolic profiles of *D. magna* exposed to (A) bisphenol A (BPA), (B) bisphenol B (BPF), and (C) bisphenol S (BPS). Averaged PCA scores are presented with their associated standard error. Statistically significant separation from the control group ($p \leq 0.05$) is indicated with an asterisk (*) close to the exposure group symbol.

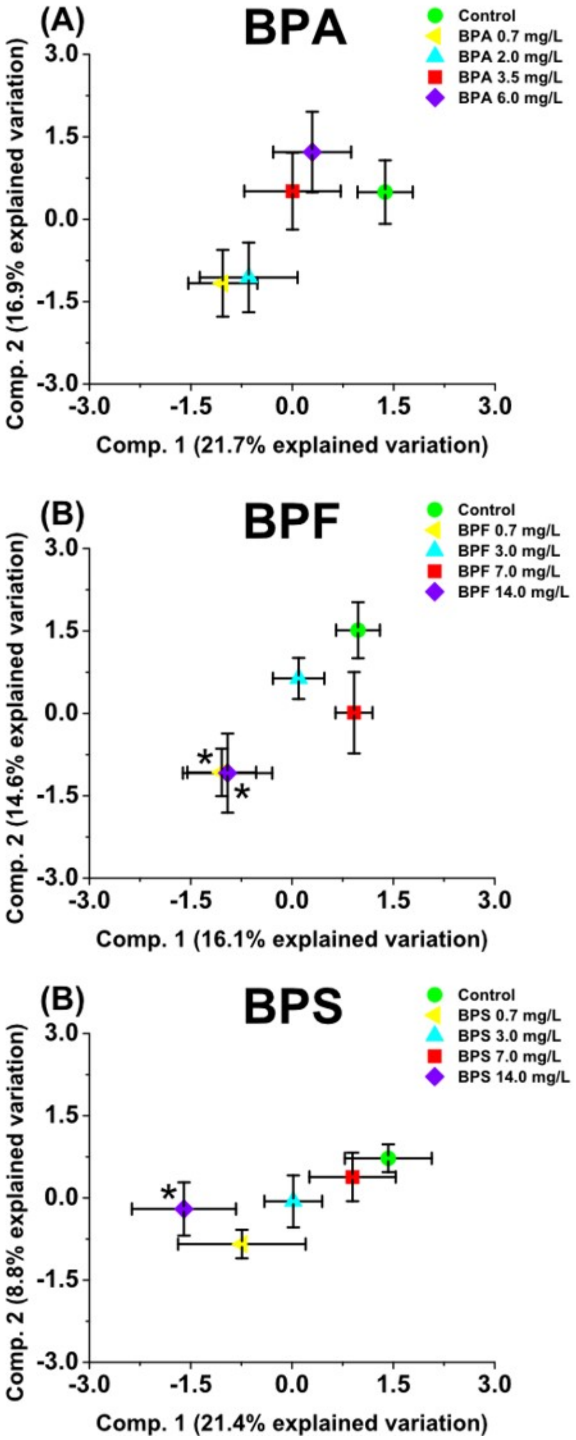

Figure S2*:* Averaged partial least square-discriminant analysis (PLS-DA) score plots (Comp. 1 vs. Comp. 3) of metabolic profiles of *D. magna* exposed to (A) bisphenol A (BPA), (B) bisphenol B (BPF), and (C) bisphenol S (BPS). Averaged PCA scores are presented with their associated standard error. Statistically significant separation from the control group ($p \leq 0.05$) is indicated with an asterisk (*) close to the exposure group symbol.

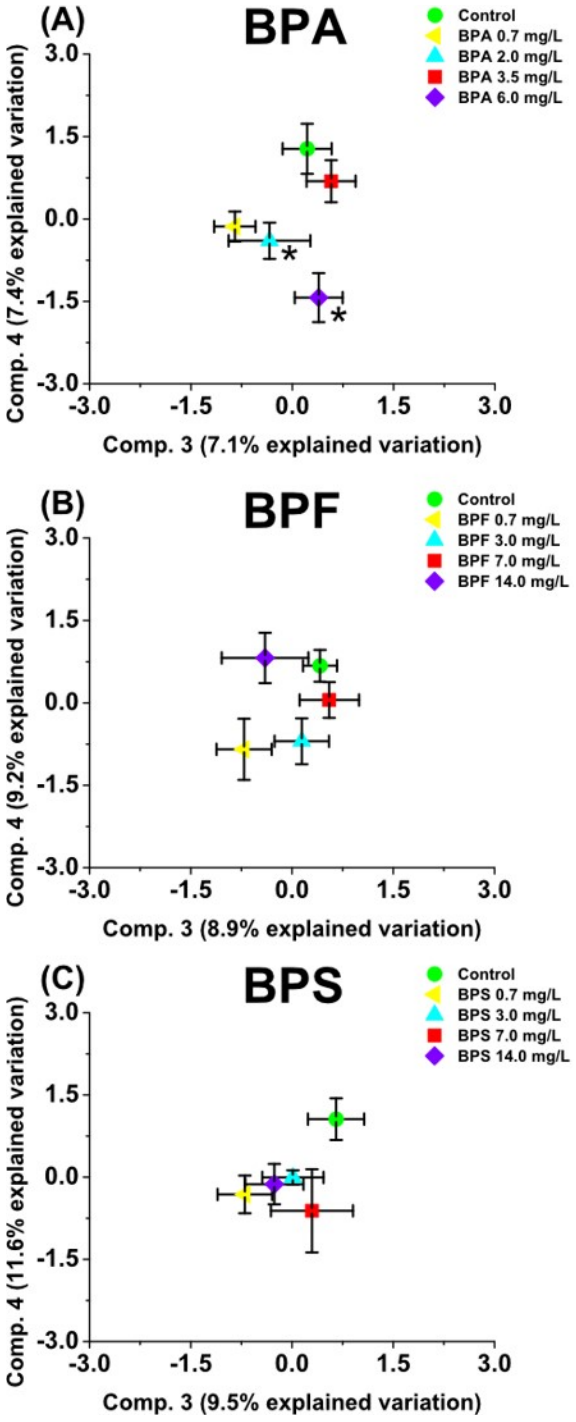

Figure S3: Averaged partial least square-discriminant analysis (PLS-DA) score plots (Comp. 3 vs. Comp. 4) of metabolic profiles of *D. magna* exposed to (A) bisphenol A (BPA), (B) bisphenol B (BPF), and (C) bisphenol S (BPS). Averaged PCA scores are presented with their associated standard error. Statistically significant separation from the control group ($p \leq 0.05$) is indicated with an asterisk (*) close to the exposure group symbol.

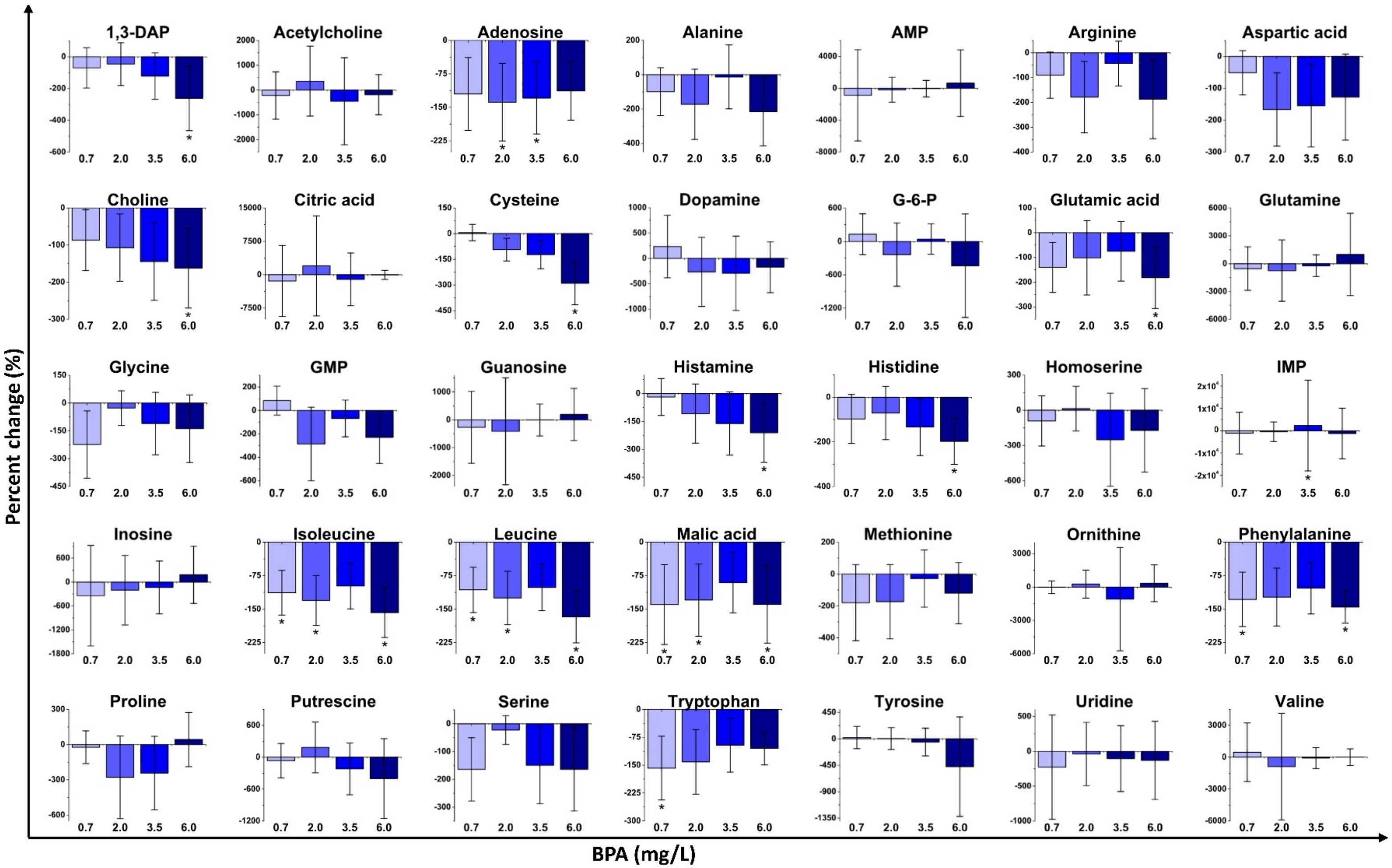

Figure S4: Metabolite percent changes of *D. magna* exposed to bisphenol A (BPA) at fractions of median effective concentration (EC_{50}) values. The percent changes of the contaminant exposure are relative to a control. (n = 10 and *$p \leq 0.05$). 1,3-DAP = 1,3-diaminopropane, AMP = adenosine monophosphate, G-6-P = glucose-6-monophosphate, GMP = guanosine monophosphate, IMP = inosine monophosphate

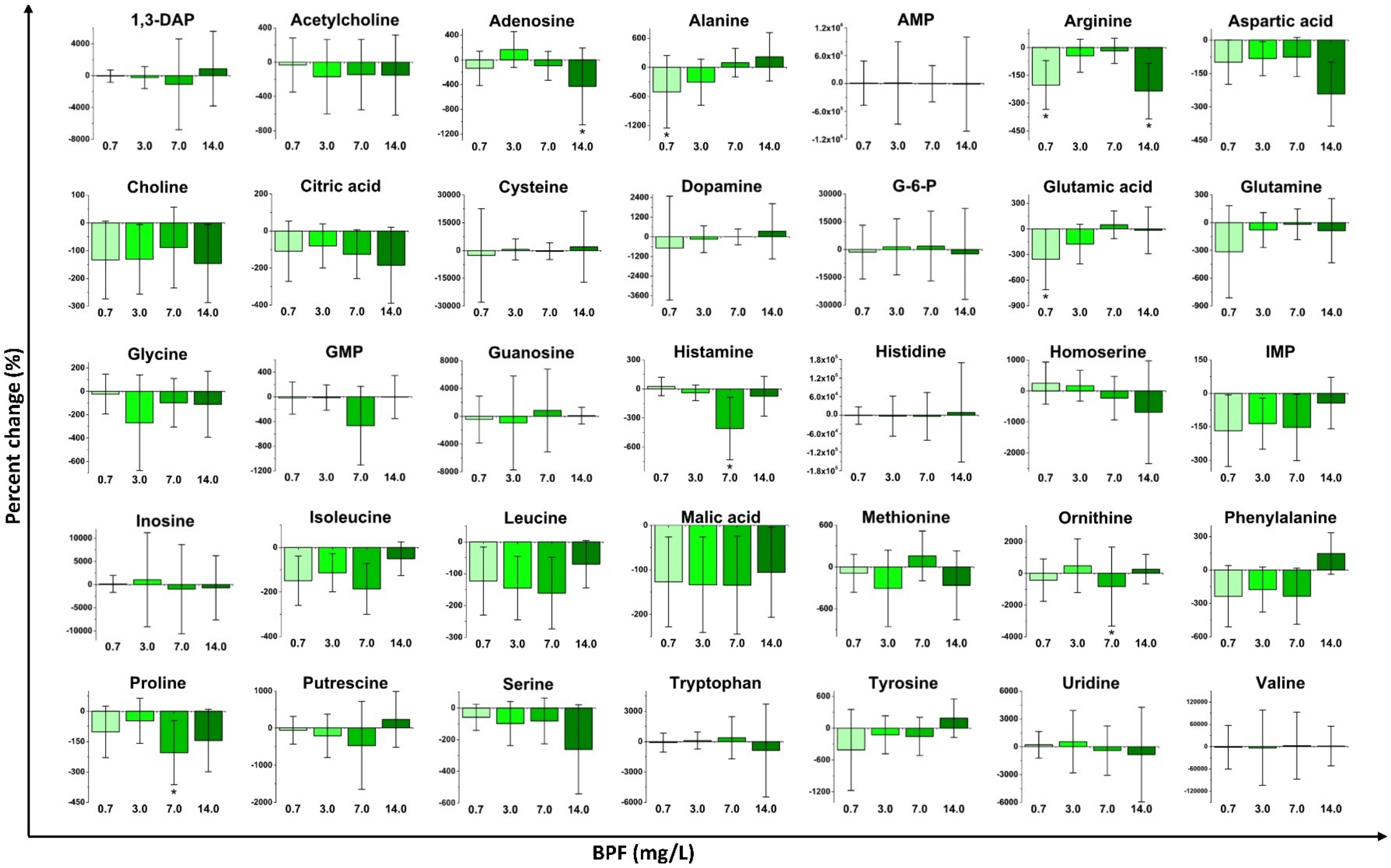

Figure S5: Metabolite percent changes of *D. magna* exposed to bisphenol F (BPF) at fractions of median effective concentration (EC_{50}) values. The percent changes of the contaminant exposure are relative to a control. (n = 10 and *p ≤ 0.05). 1,3-DAP = 1,3-diaminopropane, AMP = adenosine monophosphate, G-6-P = glucose-6-monophosphate, GMP = guanosine monophosphate, IMP = inosine monophosphate.

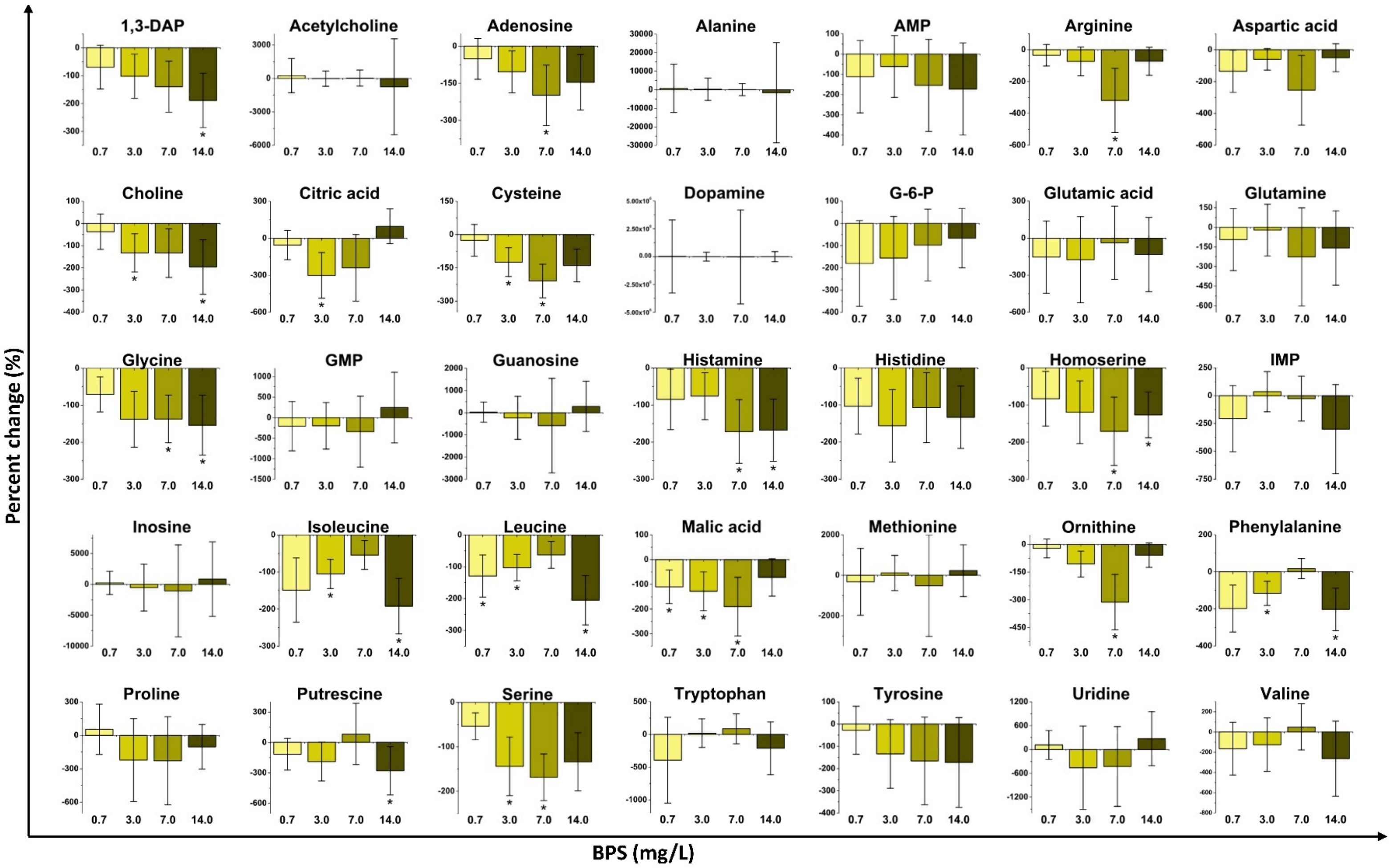

Figure S6: Metabolite percent changes of *D. magna* exposed to bisphenol S (BPS) at fractions of median effective concentration (EC_{50}) values. The percent changes of the contaminant exposure are relative to a control. (n = 10 and *p ≤ 0.05). 1,3-DAP = 1,3-diaminopropane, AMP = adenosine monophosphate, G-6-P = glucose-6-monophosphate, GMP = guanosine monophosphate, IMP = inosine monophosphate

Table S5: MetaboAnalyst evaluation for major biochemical pathways impacted by bisphenol exposure. Pathway in **red**: common to all three bisphenols (1). Pathways in **blue**: common between BPA and BPS (6). Pathways in **green**: common between BPA and BPF (4). Pathways in **purple**: common between BPF and BPS (2). Pathways in **black** – unique pathways

Compound	Exposure Concentration (mg/L)	Pathways	Hits	Metabolites associated	p-value
Bisphenol A (BPA)	0.7	Aminoacyl-tRNA biosynthesis	4/48	Phenylalanine, Isoleucine, Leucine, Tryptophan	1.77E-05
		Valine, leucine, and isoleucine biosynthesis	2/8	Leucine, Isoleucine	4.90E-04
		Valine, leucine, and isoleucine degradation	2/38	Leucine, Isoleucine	1.16E-02
		Phenylalanine, tyrosine, and tryptophan biosynthesis	1/4	Phenylalanine	1.87E-02
		Phenylalanine metabolism	1/7	Phenylalanine	3.25E-02
	2.0	Valine, leucine, and isoleucine biosynthesis	2/8	Leucine, Isoleucine	2.95E-04
		Valine, leucine, and isoleucine degradation	2/38	Leucine, Isoleucine	7.13E-03
		Aminoacyl-tRNA biosynthesis	2/48	Leucine, Isoleucine	1.13E-02
	3.5	Purine metabolism	2/63	Adenosine, IMP	3.45E-03
	6.0	Aminoacyl-tRNA biosynthesis	6/48	Histidine, Phenylalanine, Cysteine, Leucine, Isoleucine, Glutamic acid	1.13E-06
		Valine, leucine, and isoleucine biosynthesis	2/8	Leucine, Isoleucine	2.16E-03
		Histidine metabolism	2/9	Histidine, Histamine	2.77E-03
		Glyoxylate and dicarboxylate metabolism	2/24	Malic acid, Glutamic acid	1.97E-02
		Glutathione metabolism	2/26	Glutamic acid, Cysteine	2.29E-02
		Glycine, serine, and threonine metabolism	2/30	Choline, Cysteine	3.01E-02
		Phenylalanine, tyrosine, and tryptophan biosynthesis	1/4	Phenylalanine	3.71E-02
		Nitrogen metabolism	1/5	Glutamic acid	4.62E-02
		D-Glutamine and D-glutamate metabolism	1/5	Glutamic acid	4.62E-02
		Valine, leucine, and isoleucine degradation	2/38	Leucine, Isoleucine	4.67E-02
Bisphenol F (BPF)	0.7	Aminoacyl-tRNA biosynthesis	3/48	Arginine, Alanine, Glutamic acid	8.64E-05
		Arginine biosynthesis	2/12	Glutamic acid, Arginine	3.48E-04

		Alanine, aspartate, and glutamate metabolism	2/23	Alanine, Glutamic acid	1.32E-03
		**Arginine and proline metabolism**	2/31	Alanine, Glutamic acid	2.42E-03
		**Nitrogen metabolism**	1/5	Glutamic acid	1.40E-02
		**D-Glutamine and D-glutamate metabolism**	1/5	Glutamic acid	1.40E-02
		Butanoate metabolism	1/14	Glutamic acid	3.90E-02
	**3.0**	-	-	-	-
	**7.0**	**Arginine and proline metabolism**	2/31	Proline, Ornithine	2.42E-03
		**Histidine metabolism**	1/9	Histamine	2.52E-02
		**Arginine biosynthesis**	1/12	Ornithine	3.35E-02
	**14.0**	**Arginine biosynthesis**	1/12	Arginine	2.24E-02
		**Arginine and proline metabolism**	1/31	Arginine	5.74E-02
		**Aminoacyl-tRNA biosynthesis**	1/48	Arginine	8.82E-02
		**Purine metabolism**	1/63	Adenosine	1.15E-01
**Bisphenol S (BPS)**	**0.7**	**Valine, leucine, and isoleucine biosynthesis**	1/8	Leucine	1.50E-02
		Citrate cycle (TCA cycle)	1/20	Malic acid	3.73E-02
		Pyruvate metabolism	1/22	Malic acid	4.09E-02
		**Glyoxylate and dicarboxylate metabolism**	1/24	Malic acid	4.46E-02
	**3.0**	**Aminoacyl-tRNA biosynthesis**	4/48	Phenylalanine, Cysteine, Isoleucine, Leucine	2.24E-04
		**Valine, leucine, and isoleucine biosynthesis**	2/8	Leucine, Isoleucine	1.36E-03
		Citrate cycle (TCA cycle)	2/20	Malic acid, Citric acid	8.79E-03
		**Glyoxylate and dicarboxylate metabolism**	2/24	Malic acid, Citric acid	1.26E-02
		**Glycine, serine, and threonine metabolism**	2/30	Choline, Cysteine	1.94E-02
		**Phenylalanine, tyrosine, and tryptophan biosynthesis**	1/4	Phenylalanine	2.98E-02
		**Valine, leucine, and isoleucine degradation**	2/38	Leucine, Isoleucine	3.04E-02
	**7.0**	**Glutathione metabolism**	3/26	Glycine, Cysteine, Ornithine	9.89E-04
		**Arginine biosynthesis**	2/12	Arginine, Ornithine	4.02E-03
		**Aminoacyl-tRNA biosynthesis**	3/48	Arginine, Cysteine, Glycine	5.98E-03
		**Glyoxylate and dicarboxylate metabolism**	2/24	Malic acid, Citric acid	1.59E-02
		**Glycine, serine, and threonine metabolism**	2/30	Glycine, Cysteine	2.45E-02
		**Arginine and proline metabolism**	2/31	Arginine, Ornithine	2.60E-02

14.0	**Aminoacyl-tRNA biosynthesis**	4/48	Phenylalanine, Glycine, Isoleucine, Leucine	3.90E-04
	**Valine, leucine, and isoleucine biosynthesis**	2/8	Leucine, Isoleucine	1.74E-03
	**Glutathione metabolism**	2/26	Glycine, Putrescine	1.86E-02
	**Glycine, serine, and threonine metabolism**	2/30	Choline, Glycine	2.45E-02
	**Phenylalanine, tyrosine, and tryptophan biosynthesis**	1/4	Phenylalanine	3.35E-02
	**Valine, leucine, and isoleucine degradation**	2/38	Leucine, Isoleucine	3.82E-02

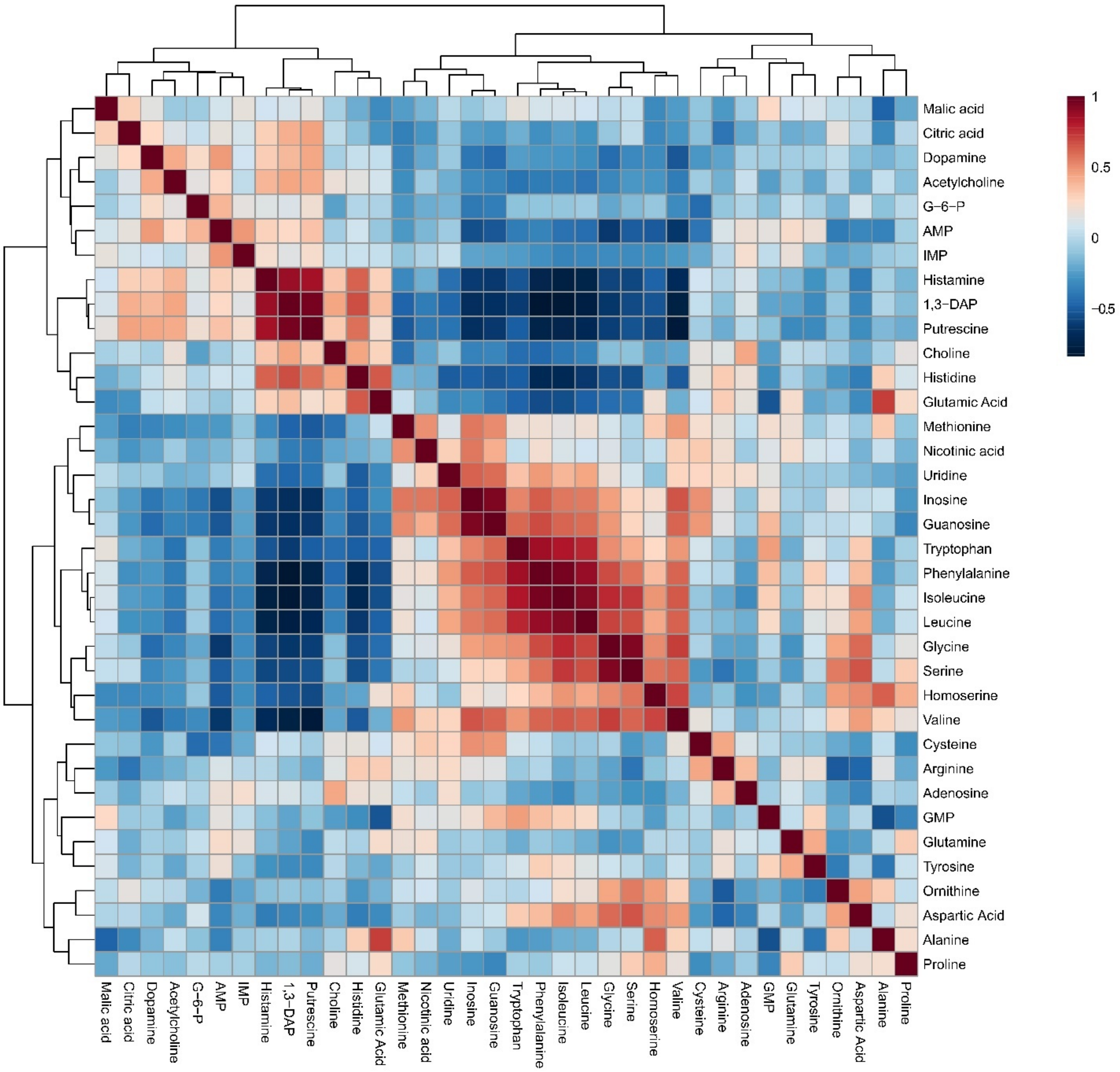

Figure S7: Pearson correlation heatmap for bisphenol A (BPA) exposure which demonstrates the correlation between metabolites. Dark red indicates a strong positive correlation and dark blue represents a strong negative correlation between the two given metabolites. 1,3-DAP = 1,3-diaminopropane, AMP = adenosine monophosphate, G-6-P = glucose-6-monophosphate, GMP = guanosine monophosphate, IMP = inosine monophosphate.

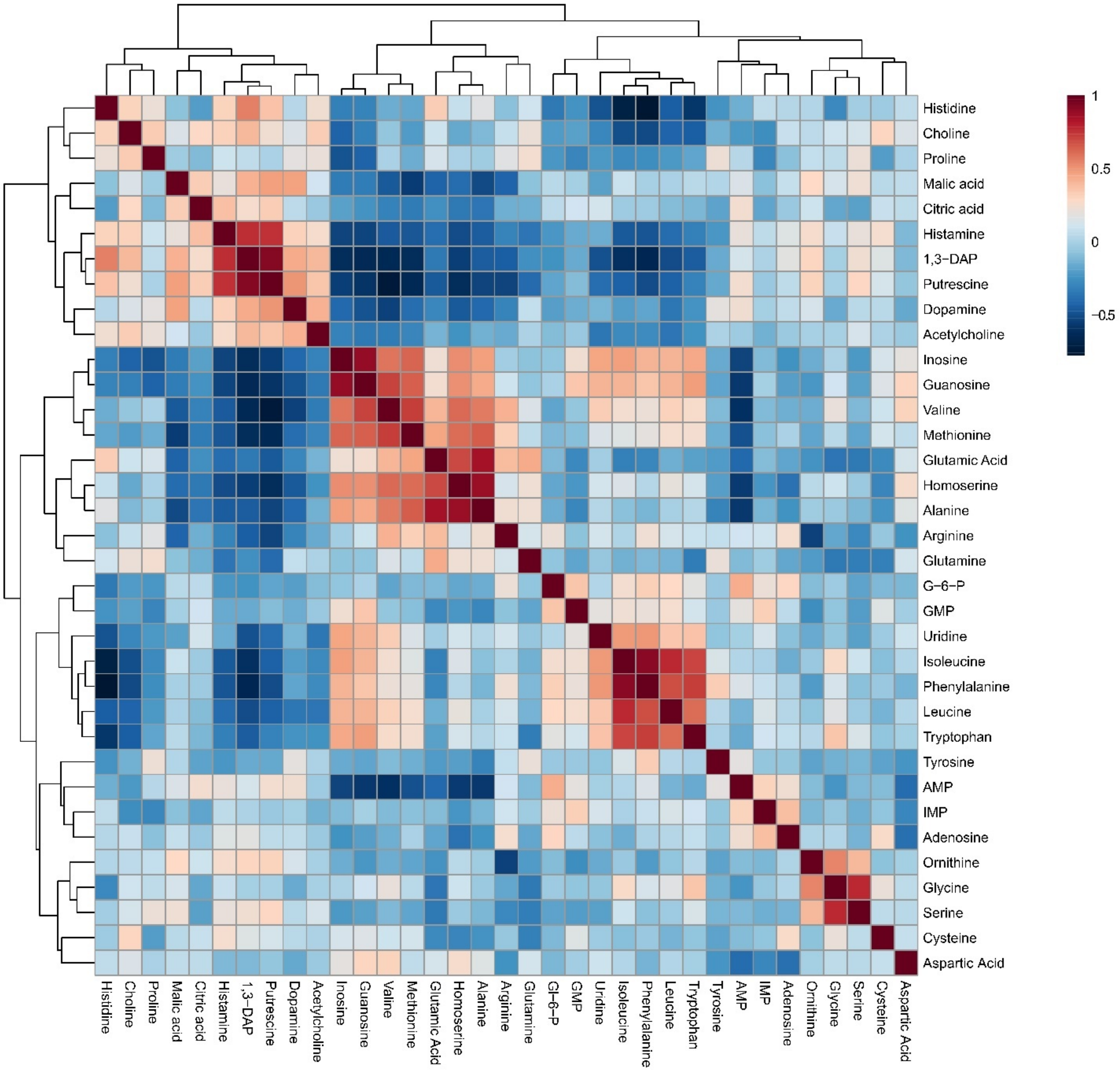

Figure S8: Pearson correlation heatmap for bisphenol F (BPF) exposure which demonstrates the correlation between metabolites. Dark red indicates a strong positive correlation and dark blue represents a strong negative correlation between the two given metabolites. 1,3-DAP = 1,3-diaminopropane, AMP = adenosine monophosphate, G-6-P = glucose-6-monophosphate, GMP = guanosine monophosphate, IMP = inosine monophosphate.

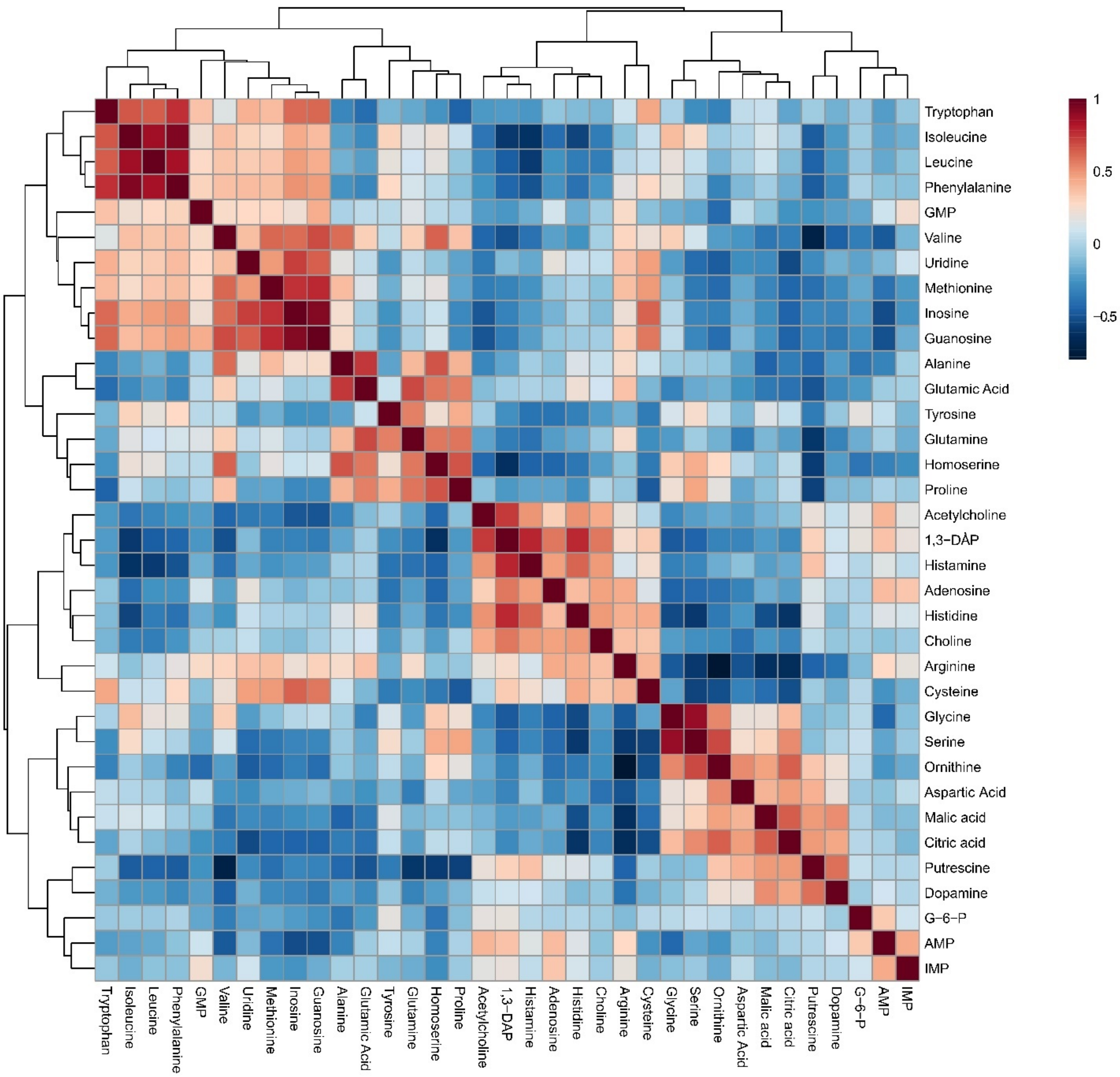

Figure S9: Pearson correlation heatmap for bisphenol S (BPS) exposure which demonstrates the correlation between metabolites. Dark red indicates a strong positive correlation and dark blue represents a strong negative correlation between the two given metabolites. 1,3-DAP = 1,3-diaminopropane, AMP = adenosine monophosphate, G-6-P = glucose-6-monophosphate, GMP = guanosine monophosphate, IMP = inosine monophosphate.

References

1. Nagato, E.G.; Simpson, A.J.; Simpson, M.J. Metabolomics reveals energetic impairments in *Daphnia magna* exposed to diazinon, malathion and bisphenol-A. *Aquat. Toxicol.* **2016**, *170*, 175–186, doi:10.1016/j.aquatox.2015.11.023.

2. Chen, M.Y.; Ike, M.; Fujita, M. Acute toxicity, mutagenicity, and estrogenicity of bisphenol-A and other bisphenols. *Environ. Toxicol.* **2002**, *17*, 80–86, doi:10.1002/tox.10035.

3. Wang, Q.; Chen, M.; Shan, G.; Chen, P.; Cui, S.; Yi, S.; Zhu, L. Bioaccumulation and biomagnification of emerging bisphenol analogues in aquatic organisms from Taihu Lake, China. *Sci. Total Environ.* **2017**, *598*, 814–820, doi:10.1016/j.scitotenv.2017.04.167.

4. Rezaee, M.; Yamini, Y.; Shariati, S.; Esrafili, A.; Shamsipur, M. Dispersive liquid–liquid microextraction combined with high-performance liquid chromatography-UV detection as a very simple, rapid and sensitive method for the determination of bisphenol A in water samples. *J. Chromatogr. A* **2009**, *1216*, 1511–1514, doi:10.1016/j.chroma.2008.12.091.
